# Dual roles of myocardial mitochondrial AKT on diabetic cardiomyopathy and whole body metabolism

**DOI:** 10.1186/s12933-023-02020-1

**Published:** 2023-10-27

**Authors:** Yu-Han Chen, Albert P. Ta, Yumay Chen, Hsiao-Chen Lee, Wenjun Fan, Phang-Lang Chen, Maria C. Jordan, Kenneth P. Roos, Grant R. MacGregor, Qin Yang, Robert A. Edwards, Junfeng Li, Ping H. Wang

**Affiliations:** 1 Department of Diabetes, Endocrinology, and Metabolism, City of Hope National Medical Center, Room 1011, Gonda South Rm 1011, 1500 E. Duarte Rd., Duarte, CA 91010-3000 USA; 2 Department of Physiology and Biophysics, University of California Irvine, Irvine, CA USA; 3 Department of Biological Chemistry, University of California Irvine, Irvine, CA USA; 4 Department of Medicine, University of California Irvine, Irvine, CA USA; 5 Department of Plastic Surgery, Kaohsiung Medical University, Kaohsiung, Taiwan; 6 Department of Physiology, David Geffen School of Medicine, University of California Los Angeles, Los Angeles, CA USA; 7 Department of Developmental and Cell Biology, University of California Irvine, Irvine, CA USA; 8 Department of Pathology and Laboratory Medicine, University of California Irvine, Irvine, CA USA

**Keywords:** Mitochondrial AKT, Heart failure, ATP synthase, Mitochondria dysfunction, Diabetic cardiomyopathy, Fatty liver, Obesity

## Abstract

**Background:**

The PI3K/AKT pathway transduces the majority of the metabolic actions of insulin. In addition to cytosolic targets, insulin-stimulated phospho-AKT also translocates to mitochondria in the myocardium. Mouse models of diabetes exhibit impaired mitochondrial AKT signaling but the implications of this on cardiac structure and function is unknown. We hypothesized that loss of mitochondrial AKT signaling is a critical step in cardiomyopathy and reduces cardiac oxidative phosphorylation.

**Methods:**

To focus our investigation on the pathophysiological consequences of this mitochondrial signaling pathway, we generated transgenic mouse models of cardiac-specific, mitochondria-targeting, dominant negative AKT1 (CAMDAKT) and constitutively active AKT1 expression (CAMCAKT). Myocardial structure and function were examined using echocardiography, histology, and biochemical assays. We further investigated the underlying effects of mitochondrial AKT1 on mitochondrial structure and function, its interaction with ATP synthase, and explored in vivo metabolism beyond the heart.

**Results:**

Upon induction of dominant negative mitochondrial AKT1, CAMDAKT mice developed cardiac fibrosis accompanied by left ventricular hypertrophy and dysfunction. Cardiac mitochondrial oxidative phosphorylation efficiency and ATP content were reduced, mitochondrial cristae structure was lost, and ATP synthase structure was compromised. Conversely, CAMCAKT mice were protected against development of diabetic cardiomyopathy when challenged with a high calorie diet. Activation of mitochondrial AKT1 protected cardiac function and increased fatty acid uptake in myocardium. In addition, total energy expenditure was increased in CAMCAKT mice, accompanied by reduced adiposity and reduced development of fatty liver.

**Conclusion:**

CAMDAKT mice modeled the effects of impaired mitochondrial signaling which occurs in the diabetic myocardium. Disruption of this pathway is a key step in the development of cardiomyopathy. Activation of mitochondrial AKT1 in CAMCAKT had a protective role against diabetic cardiomyopathy as well as improved metabolism beyond the heart.

**Supplementary Information:**

The online version contains supplementary material available at 10.1186/s12933-023-02020-1.

## Background

While mitochondria are known to play a crucial role in myocardial function, there is still much that is not fully understood about the mechanisms involved. Cardiac muscle has high energy demands that must be met by the metabolism of fatty acids and glucose. In diabetic myocardium, metabolic dysfunction contributes to the development of heart failure and ATP deficiency is a key predictor of poor clinical outcomes in diabetic cardiomyopathy (DCM) [1, 2]. Impaired mitochondrial oxidative phosphorylation and lowered ATP synthesis rates are also evident in rodent models of diabetes [3–7]. Understanding how mitochondrial oxidative phosphorylation is dysregulated in the diabetic myocardium will help to identify potential targets that could be used to develop new strategies to improve myocardial function in diabetic patients.

One of the key regulators of myocardial oxidative phosphorylation is insulin receptor signaling. In insulin receptor KO mice, oxidative phosphorylation was decreased and ventricular dysfunction was exacerbated [8]. Although insulin receptor signaling is highly complex and interacts with a vast network of signaling molecules, the phosphatidylinositol 3-kinase (PI3K)-AKT/protein kinase B (PKB) pathway is responsible for the majority of the metabolic actions of insulin, and represents an important pathway of the insulin signaling network [9].

Our previous work established the role of AKT1 as a mediator between insulin receptors and cardiac mitochondria. AKT is a serine/threonine kinase directly downstream of PI3K, and an important mediator of the major metabolic actions of insulin and other growth factors. The signaling actions of AKT promotes cellular metabolism, growth, and survival [10–12]. While its function as a cytosolic regulator has been characterized, we found that insulin also induced the translocation of phospho-AKT1 to all mitochondrial compartments—the inner membrane, outer membrane, and intermembrane space- in cardiac muscle [13]. Conversely, in both rodent models of insulin deficiency (type 1 diabetes) and insulin resistance (type 2 diabetes), AKT1 translocation to mitochondria was impaired in the myocardium [14]. Active mitochondrial AKT1 signaling stimulated ATP production, while in AKT1 KO mice, Complex V activity was lowered [13]. Taken together, these studies have shown that mitochondrial AKT1 it is a critical link between insulin signaling and mitochondrial function.

Impaired AKT1 translocation to mitochondria is an intriguing paradigm underlying dysregulation of myocardial bioenergetics in the context of diabetes and insulin resistance. However, its pathophysiological impact in vivo are largely unknown. The purpose of the present study was to establish the causal relationship between cardiac mitochondrial AKT1 signaling and the development of DCM. To this end, we generated two inducible cardiomyocyte-specific transgenic mouse models to modulate mitochondrial AKT1 signaling with the aims of (1) characterizing the cardiomyopathy induced by inhibiting mitochondrial AKT1 signaling and (2) demonstrating that enhancing mitochondrial AKT1 signaling has a protective role against DCM.

## Methods

### Transgenic mouse models

A mitochondria-targeting dominant negative Akt1 (*mito-dnAkt1*) construct was made using strategies based on our previously published studies [15, 16]. To generate a dominant negative *Akt1*, K179 of *Akt1* cDNA was mutated to methionine to abolish ATP binding. T308 and S473 phosphorylation sites were unaltered and thus any protein interactions with AKT1 should be intact, however, their phosphorylation by AKT1 would be impaired. For mitochondria targeting, the human cytochrome c oxidase subunit 8A sequence (NP_004065.1; MSVLTPLLLRGLTGSARRLPVPRAKIHSL) was added to the 5′ end. The mitochondria targeting sequence is cleaved during mitochondrial import. A 6 × His-tag was added at the 3′ end for detection of the resultant protein.

We next cloned the *mito-dnAkt1* construct into the ROSA26 locus of C57BL/6J mice. The coding sequence was first cloned into pCALNL-dsRed [17]—a gift from Dr. Constance Cepko (Addgene, 13769)—following removal of the Ds-Red coding sequence. In pCALNL, a CMV immediate early gene enhancer and chicken β-actin gene promoter/intron and beta-globin polyA signal drives expression of the coding sequence [18, 19]. To mediate targeting to the ROSA26 (Gt(ROSA)26Sor) locus, mito-dnAkt1-pCALNL was cloned into pROSA26-1—a gift from Dr. Philippe Soriano (Addgene, 21714) [20]—with the CAG promoter oriented opposed to the ROSA26 RNA transcript. Expression of mito-dnAKT1 begins following Cre-mediated deletion of a floxed Neo-SV40-PolyA sequence between the promoter and the coding sequence [20]. JM8.N4 ES cells (derived from C57BL/6NTac mice) [21] were electroporated with linearized targeting constructs and 32 G418-resistant clones for each construct were screened for homologous recombination. Targeting efficiency was 50% for the *mito-dnAkt1* construct. Correctly targeted ES cells were microinjected into C57BL/6J blastocysts. The resulting male ROSA26-CAG-LNL-*mito-dnAKT1* chimeras (MDNAKT) were bred with C57BL/6 mice to establish the lines used in this study. Engineering of mES cells, genomic characterization, microinjection of blastocysts, and production of founder transgenic mice were carried out at the UCI Transgenic Mouse Facility [22]. MDNAKT mice were then crossed with a well-studied Cre transgenic mouse strain, Myh6-CreER^T2^ (Myh6-Cre), to generate a transgenic mouse line with inducible overexpression of mito-dnAKT1 in cardiomyocytes (CAMDAKT).

Similarly, the strategy above was utilized to generate a transgenic mouse line with inducible overexpression of cardiomyocyte-specific, constitutively active mitochondrial AKT1 (CAMCAKT). In contrast, a *mito-caAkt1* construct was used with *Akt1* cDNA containing threonine T308 and serine S473 to glutamic acid phosphomimetic mutations.

Mice were kept in a temperature-controlled environment and fed ad libitum with laboratory chow (Envigo 2020X, Teklad). Cre-mediated recombination was achieved by administration of Tamoxifen (TAM), 40 mg/kg body weight/day i.p. one time at 8 weeks of age. Corn oil was injected as a vehicle control. Expression of the transgene was detected 10 h after first injection and verified 4 weeks afterwards. The stability of the His-tagged mutant AKT1 protein was demonstrated in our previous study [15]. Experiments were primarily performed in male mice as female mice are more resistant to cardiomyopathy due to protective factors such as estrogen [23]. In selective experiments, female mice were used to confirm the phenotype.

### High fat diet studies

To generate a diabetes phenotype, mice were placed on a high fat chow and fructose water diet (HFFD). The high fat chow contained 45% calories from fat (TD.08811, Envigo Teklad Custom Diet, UK). Cage water was replaced with a 30% fructose solution (Sigma F0127-5KG). The length of HFFD is specified per given experiment. Chow and water were replaced at 2-week intervals. Male mice were studied due to their susceptibility to diet induced metabolic changes [24]. Previous metabolic studies found female mice to be more resistant to weight gain, insulin insensitivity and cardiomyopathy [25].

### Statistical analysis

Data were analyzed with GraphPad Prism 9 software and presented as mean ± SEM. Unpaired student’s T test was used to determine statistical significance. P values < 0.05 were considered statistically significant and notated as: *(p < 0.05), **(p < 0.01), ***(p < 0.001), ****(p < 0.0001).

Additional experimental methods and materials are provided in the Additional file 2.

Supplementary figures are included in the Additional file 1.

## Results

### CAMDAKT transgenic mice expressed cardiomyocyte-specific mitochondria-targeted dominant negative AKT1

**Fig. 1:**
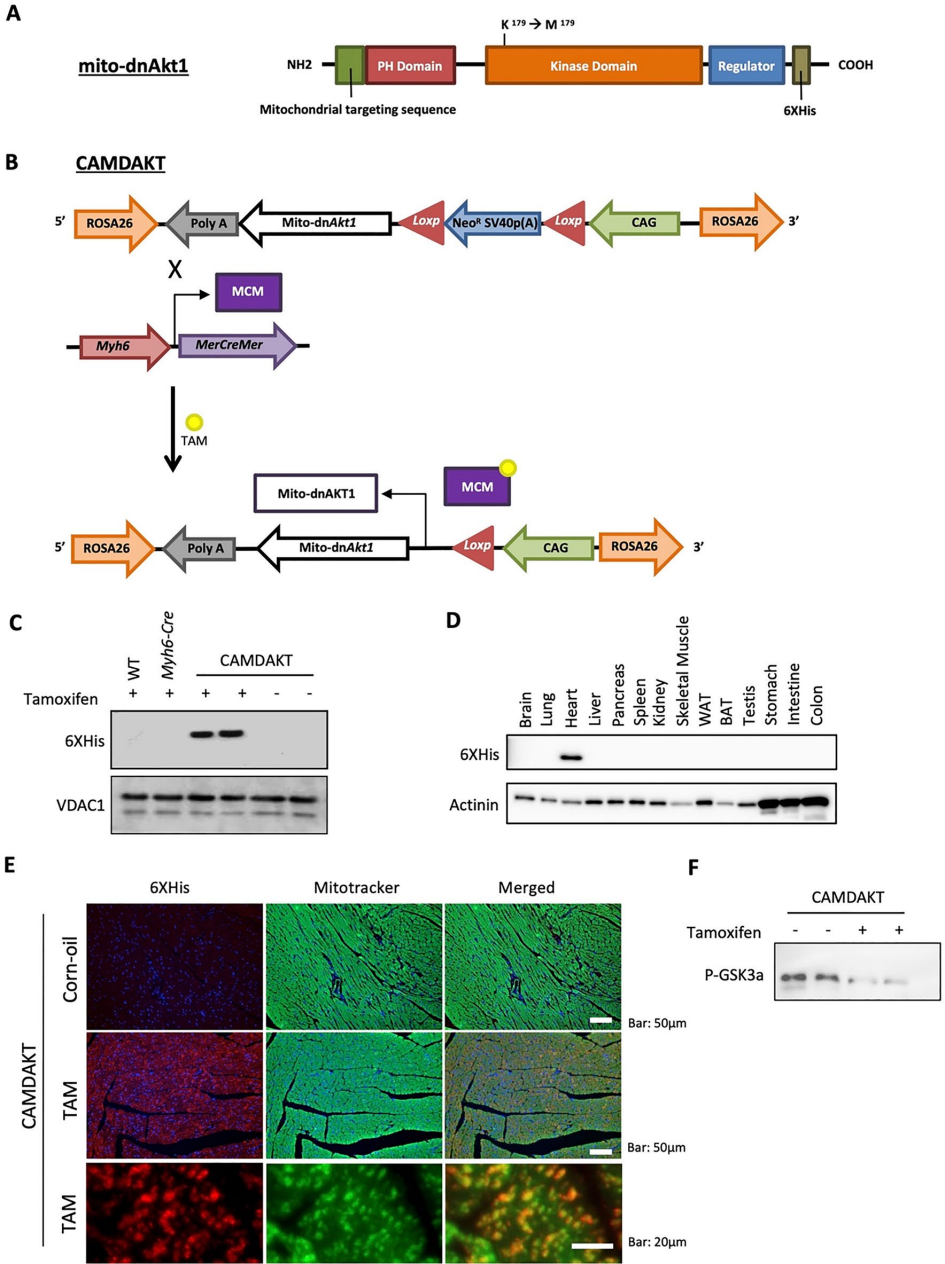
Generation of transgenic mice with inducible cardiomyocyte-specific expression of mitochondria-targeting dominant negative AKT1. **A** The *mito-dnAkt1* construct contains a dominant negative AKT1 with a mitochondria localization sequence and His-tag. **B** Scheme of model to overexpress cardiac-specific mitochondria-targeting dominant negative AKT1 (CAMDAKT). Transgenic mice harboring *mito-dnAkt1* were crossed with Myh6-Cre mice (Cre recombinase expressed in cardiomyocytes) to generate CAMDAKT bi-genic mice. The Neo cassette, containing Neo cDNA followed by an SV40 PolyA signal to terminate transcription, was removed by Cre-mediated recombination upon injection with tamoxifen (TAM). **C** Mitochondria specific expression of mito-dnAKT1 in CAMDAKT heart. Eight-week-old CAMDAKT mice were injected with TAM or vehicle-corn oil (CO). 10 h after injection, the mitochondrial fraction was isolated and expression of mito-dnAKT1 was analyzed by western blotting. The mito-dnAKT1 protein, detected by 6x-His antibodies, was expressed in cardiomyocyte mitochondria isolated from TAM-CAMDAKT mice but not in CO-CAMDAKT mice, TAM-Myh6-Cre mice or TAM-wildtype (WT) mice. VDAC1 was used as a loading control and mitochondria marker. **D** Cardiac specific expression in CAMDAKT mice after TAM induction. Total protein was isolated from the specified organs of CAMDAKT mice 10 h after TAM injection. Tamoxifen-induced mito-dnAKT1 protein expression was detected by 6x-His antibodies in the heart specifically and not detected in other organs examined. Actinin was used as a loading control. **E** Mitochondria specific localization of mito-dnAKT1. Co-localization of mito-dnAKT1 and mitochondria was visualized by immunostaining. 24 h after injection with TAM, hearts were fixed and embedded with paraffin. 4-micron thin sections were immunostained with 6x-His antibodies (red) followed by staining with MitoTracker™ Green FM (green) and DAPI (blue). Mito-dnAKT1was detected in TAM-CAMDAKT cardiomyocytes and colocalized with mitochondria. Scale bar = 50 μm (upper), 20 μm (lower). **F** Inactivation of AKT activity in CAMDAKT cardiac mitochondria. AKT activity in the mitochondrial protein fractions was analyzed by an in vitro kinase assay using recombinant GSK3α as substrate, 24 h after TAM injection

To study the effects of impaired cardiac mitochondrial AKT signaling in vivo, we developed a bigenic mouse model which enabled Tamoxifen-inducible, cardiomyocyte-specific expression of mitochondria-targeted dominant negative AKT1 (CAMDAKT). Expression of dominant negative mitochondrial AKT1 mutant protein (mito-dnAKT1) competitively inhibits endogenous mitochondrial AKT1 kinase activity. CAMDAKT bigenic mice were obtained by crossing hemizygous *Myh6-Cre* mice with MDNAKT mice containing our engineered *mito-dnAkt1* transgene [15, 16]. *Myh6-Cre* mice express cardiomyocyte-specific CreER^T2^ driven by a myosin heavy chain 6 promoter. The *mito-dnAkt1* transgene uses the CAG promoter to express a dominant negative, ATP binding site-mutated, AKT1 (K179M) with a mitochondria targeting sequence at the N-terminus and 6 × His-tag at the C-terminus, inserted into the *Gt(ROSA)26Sor* locus (Fig. 1A). A floxed Neo cassette with an SV40 polyA sequence was used to prevent expression of *mito-dnAkt1* prior to induction of Cre-ER^T2^ activity by TAM (Fig. 1B). This design provided temporally controlled and tissue-specific expression of mito-dnAKT1.

To validate our model, mitochondria were isolated from CAMDAKT and control mice, and mitochondrial proteins were resolved with SDS-PAGE for immunoblotting. CAMDAKT mice were injected with Tamoxifen (TAM) at 8 weeks of age to induce expression of mito-dnAKT1. Corn oil (CO) was injected as a vehicle control at the same time intervals as TAM-injected mice. Expression of mito-dnAKT1 in isolated cardiac mitochondria from TAM-CAMDAKT mice were confirmed by detection of 6 × His-tag (Fig. 1C, Additional file 1: Fig. S1A). The mutant protein was not detected in CO-CAMDAKT mice, *Myh6-Cre* mice or wildtype mice. To verify cardiac-specific expression of mito-dnAKT1, total protein were extracted from select tissues after induction of mito-dnAKT1 in TAM-CAMDAKT mice. Mito-dnAKT1 was expressed exclusively in the heart (Fig. 1D). To confirm proper translocation, myocardial sections were imaged by immunofluorescence microscopy and showed mito-dnAKT1, detected by 6x-His antibody, was colocalized to mitochondria, stained by MitoTracker™ Green FM (Fig. 1E). In a previous study, we confirmed that this AKT1 targeting strategy localized mito-dnAKT1 to mitochondria, but not to the nucleus nor cytosol and did not alter cytosolic nor nuclear AKT1 activity [13, 16]. Kinase activity of mitochondrial AKT1 were analyzed with recombinant GSK3α to confirm the dominant negative effect of TAM-CAMDAKT in the cardiac mitochondria and showed cardiomyocyte mitochondrial AKT1 activity was significantly suppressed 24 h after induction (Fig. 1F). These results showed that CAMDAKT mice could be used as an experimental model to study impaired mitochondrial AKT1 signaling in cardiac muscle.

### Inhibition of cardiac mitochondrial AKT1 signaling led to development of cardiomyopathy and mitochondrial dysfunction

**Fig. 2:**
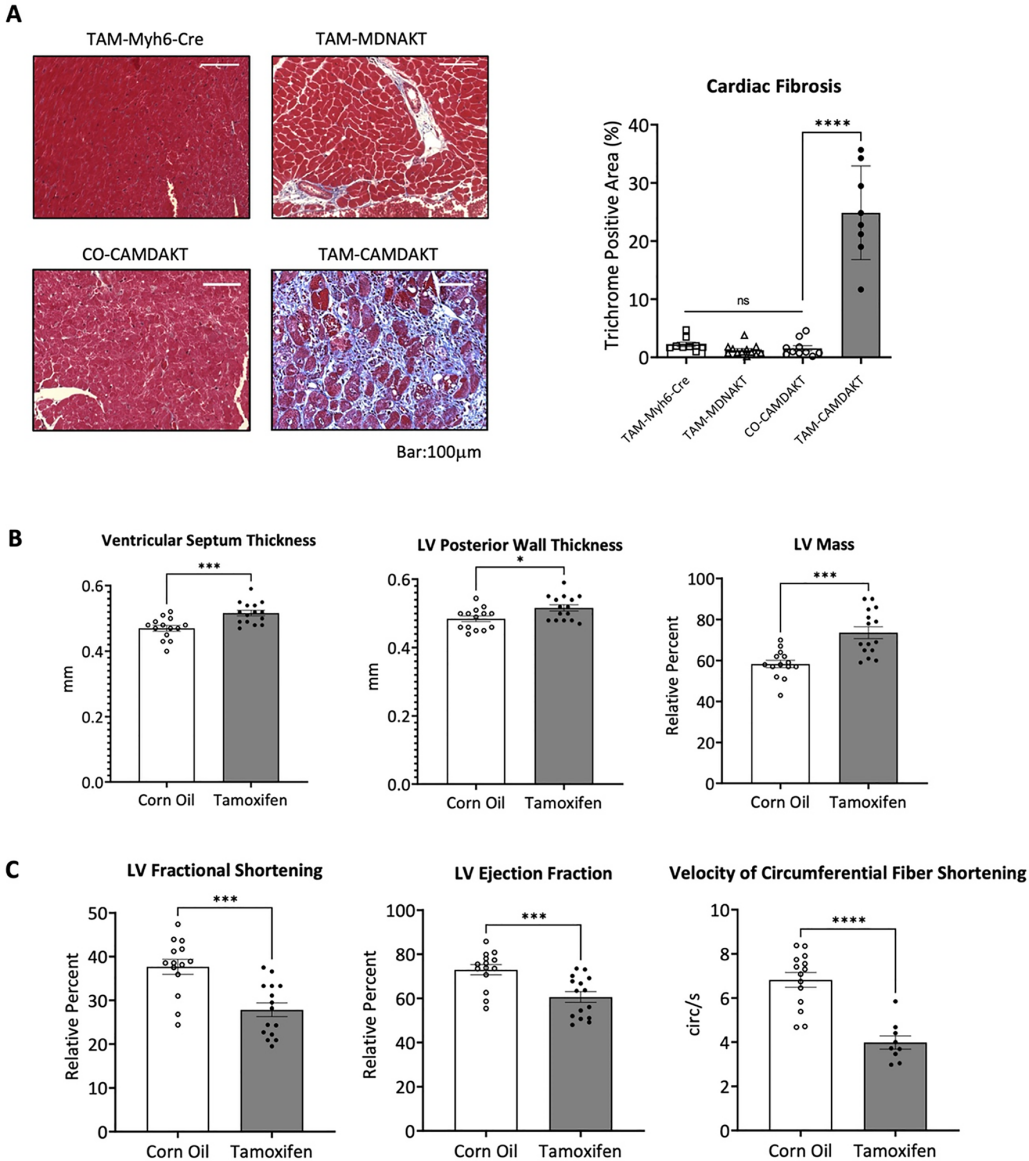

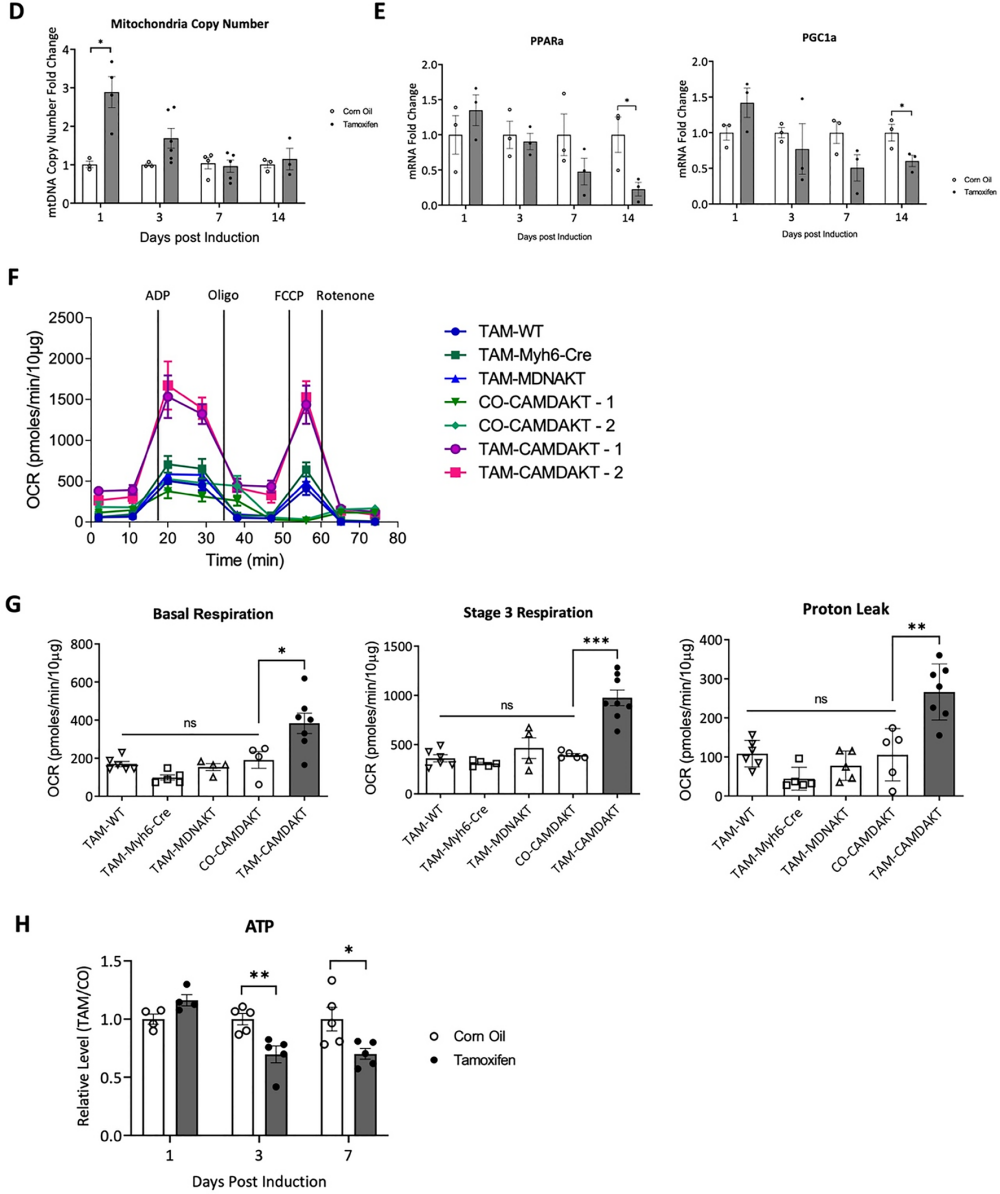
Inhibition of cardiac mitochondrial AKT1 signaling led to development of cardiomyopathy and mitochondrial dysfunction. **A** Left, representative images of trichrome stained myocardial sections. TAM or CO was administered once as indicated. 7 days after injection, cardiac fibrosis was quantified by trichrome staining. Scale bar = 100 μm. Right, quantification of cardiac fibrosis (n = 12 (TAM-Myh6-Cre), n = 9 (TAM-MDNAKT), n = 10 (CO-CAMDAKT), n = 8 (TAM-CAMDAKT); p < 0.0001). **B** Cardiac left ventricular septum thickness, posterior wall thickness and ventricular mass were analyzed by echocardiogram 7 days after TAM or CO injection in CAMDAKT mice. LV Mass is given as a relative percent of total heart mass. (n = 14 (CO), n = 15 (TAM); p < 0.001, < 0.05, < 0.001). **C** Cardiac function was measured by echocardiogram 7 days after TAM or CO injection in CAMDAKT mice. Left ventricular fractional shortening, ejection fraction and velocity of circumferential fiber shortening were quantified (n = 14 (CO), n = 15 (TAM); p < 0.001, < 0.001, < 0.0001). **D** Mitochondrial DNA were analyzed by RT-qPCR and presented as a ratio of mitochondrial DNA to nuclear DNA fold change relative to CO-CAMDAKT (n = 3 (CO), n = 3–6 (TAM); p < 0.05). **E** Expression of mitochondrial biogenesis markers PPARα and PGC1α were measured by RT-qPCR at timepoints post TAM or CO injection. Data are presented as relative fold change to CO-CAMDAKT (n = 3 per group; p < 0.05, < 0.05). **F** Representative graph of mitochondrial respiration, measured by Seahorse assay, 3 days after TAM or CO injection. **G** Basal respiration, stage 3 respiration, and proton leak in mitochondria were calculated. Myocardial mitochondria were isolated from TAM-wild type (n = 6), TAM–Myh6-Cre (n = 5), TAM-MDNAKT (n = 5), CO-CAMDAKT (n = 5) and TAM–CAMDAKT (n = 7) (p < 0.05, < 0.001, < 0.01). **H** ATP levels in CAMDAKT mitochondria from 1, 3 and 7-days post TAM or CO injection. ATP levels were quantified by mass spectrometry. Data are presented as relative levels to CO-CAMDAKT (n = 4–5 per group; p < 0.01, < 0.05)

We first investigated whether inhibiting cardiac mitochondrial AKT1 could lead to cardiomyopathy. Myocardial fibrosis was observed in TAM-CAMDAKT mice 7 days post-induction. Trichrome staining revealed significant collagen deposition in TAM-CAMDAKT, 25% by area, a 16-fold increase (p < 0.0001) compared to CO-CAMDAKT. Minimal fibrosis was found in corn-oil injected CAMDAKT mice (CO-CAMDAKT), TAM-Myh6-Cre, and TAM-MDNAKT (Fig. 2A, Additional file 1: Fig. S2A). Furthermore, the heart mass of TAM-CAMDAKT mice, as a proportion of body weight, was significantly increased compared to CO-CAMDAKT. Control groups TAM-Myh6-Cre and TAM-MDNAKT were not significantly different (Additional file 1: Fig. S2B). In female mice, fibrosis was less severe- 9% collagen by area was detected in TAM-CAMDAKT, a fourfold increase (p < 0.01) over CO-CAMDAKT. Minimal collagen was detected in TAM-Myh6-Cre and TAM-MDNAKT mice (Additional file 1: Fig. S2C).

Because previous studies showed that myocardial fibrosis did not necessarily correlate with ventricular dysfunction, we next measured ventricular dimensions and function by echocardiography [26, 27]. 42 days post-induction in TAM-CAMDAKT, ventricular septal thickness, left ventricular posterior wall thickness, and left ventricular mass were all increased in TAM-CAMDAKT mice by 11%, 7.6%, and 27% respectively (p < 0.001, < 0.05, < 0.001) (Fig. 2B). Left ventricular function was diminished. In TAM-CAMDAKT mice, left ventricular fractional shortening, left ventricular ejection fraction, and the velocity of circumferential fiber shortening were lower by 26%, 17%, and 30% respectively when compared to corn oil-injected controls (p < 0.001, < 0.001, < 0.0001) (Fig. 2C). These results indicated that TAM-CAMDAKT mice had larger left ventricle mass and reduced left ventricle systolic function, and together, showed the profound detrimental effects of impaired mitochondrial AKT1 signaling.

Inhibition of cardiac mitochondrial AKT1 had an immediate effect on mitochondrial homeostasis. One day after TAM-CAMDAKT induction, mitochondrial DNA abundance was increased 61% before returning to the same level as CO-CAMDAKT at 14 days post TAM treatment, indicating an initial surge in mitochondrial abundance remodeling (p < 0.05) (Fig. 2D). Simultaneously, expression of PPARα and PGC1α, markers of mitochondrial biogenesis, may have been elevated upon induction of mito-dnAKT1 before being significantly downregulated 14 days post induction, which suggested a modulation of mitochondrial dynamics (p < 0.05, < 0.05) (Fig. 2E).

Mitochondrial function was measured after mitochondrial DNA abundance had returned to baseline 3 days after induction of TAM-CAMDAKT. Inhibition of mitochondrial AKT1 increased mitochondrial oxygen consumption (Fig. 2F). Mitochondria oxygen flux assays revealed that basal mitochondria oxygen consumption, as well as stage 3 oxygen consumption, were significantly elevated in TAM-CAMDAKT. There were no significant differences among the four control conditions: TAM-WT, TAM-Myh6-Cre, TAM-MDNAKT, and CO-CAMDAKT. Higher proton leak in the TAM-CAMDAKT mitochondria suggested an increase in uncoupled respiration (p < 0.05, p < 0.001, p < 0.01) (Fig. 2G). Despite the increase in respiration, cardiomyocyte levels of ATP were decreased at 3- and 7-days post-induction of TAM-CAMDAKT (p < 0.01, < 0.05) (Fig. 2H). Together these results demonstrated that mitochondrial AKT1 signaling was critical for maintaining mitochondrial bioenergetics and respiratory efficiency.

### Inhibition of cardiac mitochondrial AKT1 signaling led to disruption of ATP synthase complex and mitochondrial structure

**Fig. 3:**
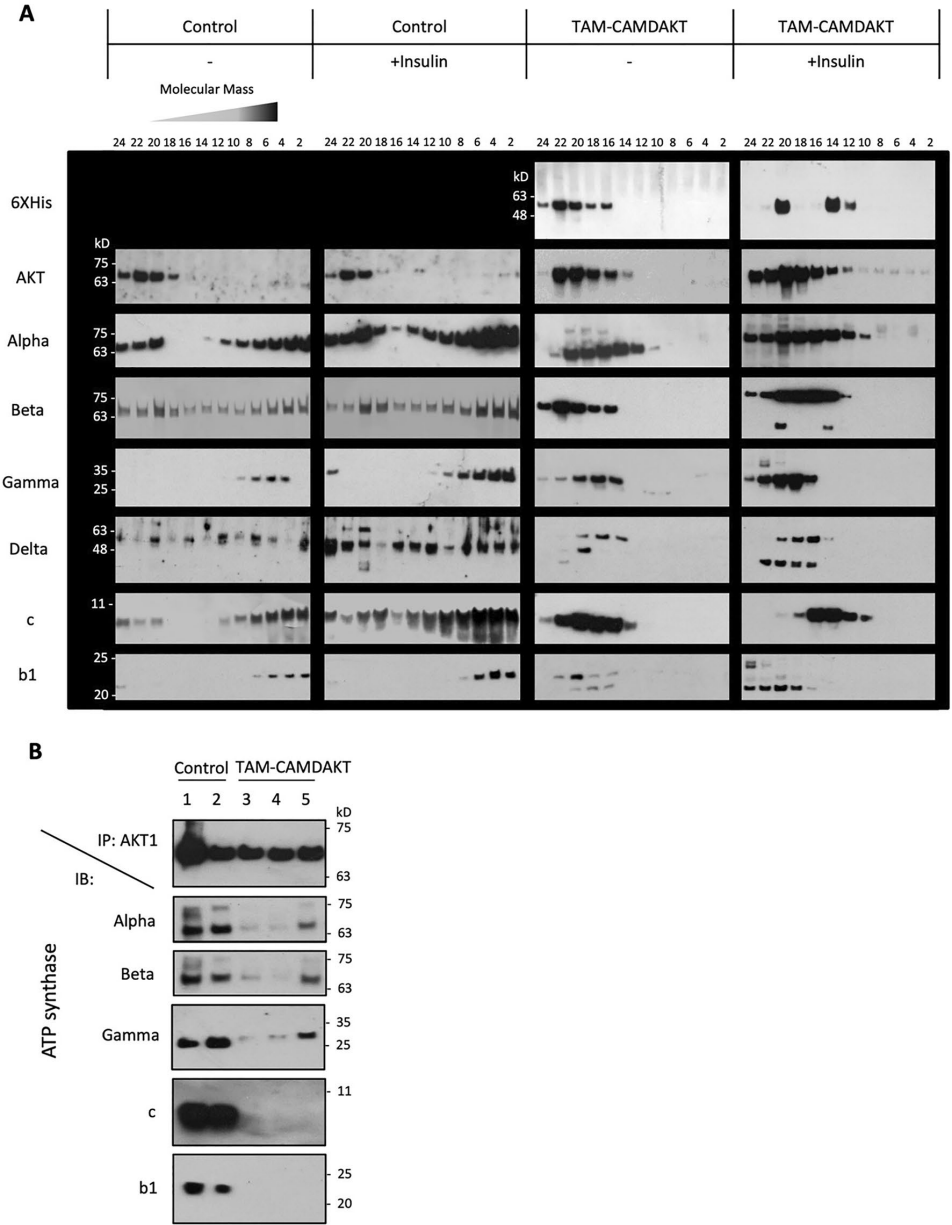

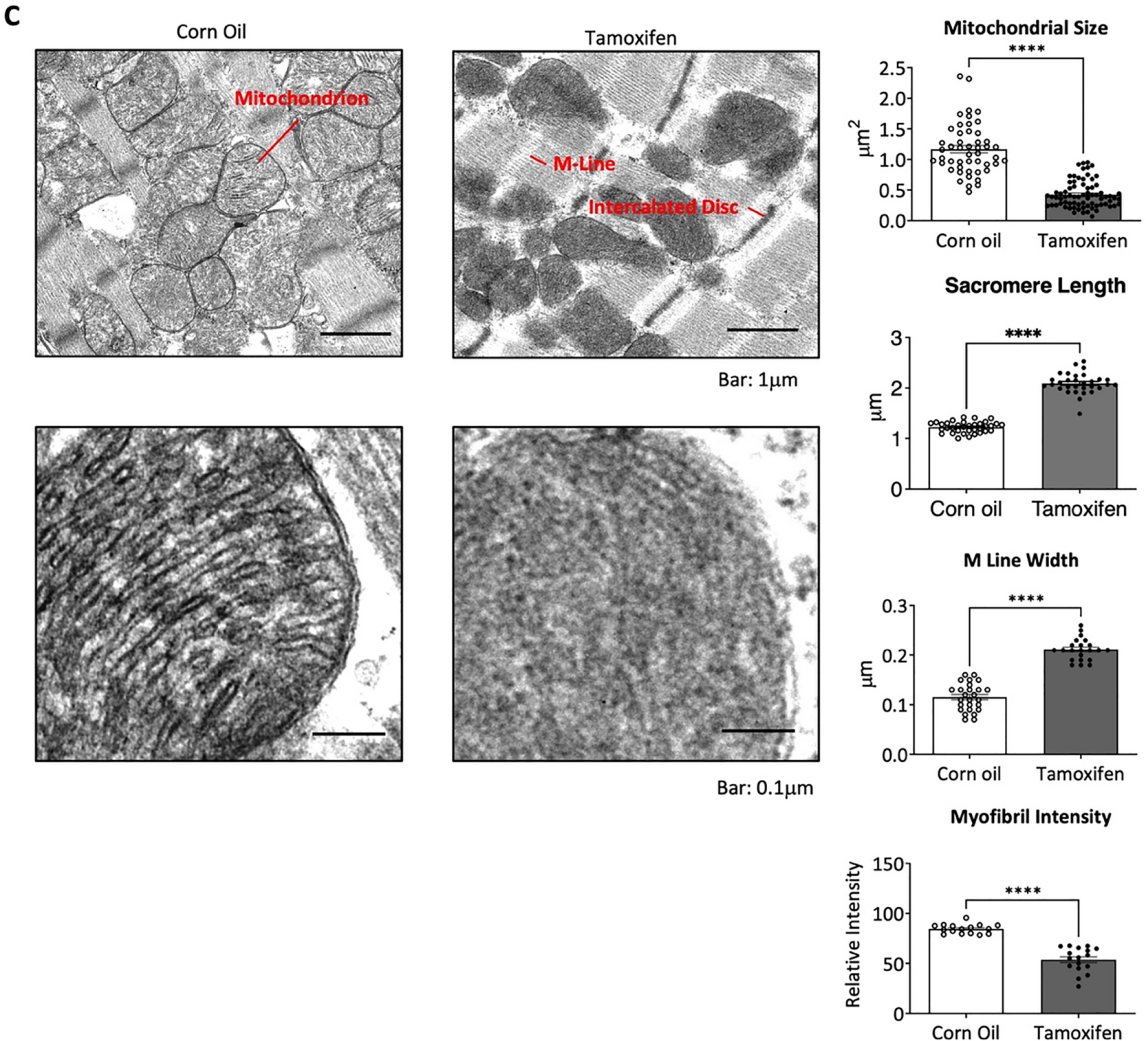
Mitochondrial AKT1 promoted ATP synthase complex integrity. **A** Interruption of the formation of large ATP synthase complexes in TAM-CAMDAKT cardiac mitochondria. A sucrose gradient was used to fractionate mitochondrial protein complexes by size. Insulin stimulation in the TAM-CAMDAKT mice could not promote larger complex assembly as seen in the control mice. Representative blots are shown. Subunits b1 and c, required for ATP synthase anchoring of the cristae, were not associated with the larger complex in the TAM-CAMDAKT heart (n = 4 each group). **B** Interaction between ATP synthase complex and AKT1. ATP synthase subunits (ATP5A) and AKT1 from isolated mitochondria were co-immunoprecipitated. Representative blots are shown. ATP synthase subunit c and b1 did not associate with the AKT1 complex, which contains ATP synthase subunit alpha and beta, in TAM-CAMDAKT mitochondria but were evident in control mitochondria. (n = 3 each group). **C** Left, representative TEM images of CAMDAKT myocardial sections, 7 days after TAM or CO injection, showed loss of cristae in TAM-CAMDAKT mice compared to CO-CAMDAKT control animals (n = 2 each group). Scale bar = 1 μm (upper), 0.1 μm (lower). Right, quantification of TEM images. Each data point was the calculated average measurements of one image (CO:12 images, TAM:15 images). Cross-sectional areas of mitochondria, length of sarcomeres, M line widths, and intensity of myofibrils, a measure of myofibril density, were quantified. (All p < 0.0001)

Mitochondrial oxidative phosphorylation complexes can exist as different masses and be composed of different subunits [28]. The bioenergetic dysfunction in TAM-CAMDAKT mice led us to investigate whether ATP synthase complex assembly was negatively impacted. After stimulating AKT1 signaling by insulin injection, mitochondria were isolated from hearts of control and TAM-CAMDAKT mice. Mitochondrial proteins were sub-fractionated using sucrose gradient centrifugation (Additional file 1: Fig. S3A). Western blotting of the sub-fractionated protein complexes from mice revealed an increase in abundance of complex V subunits in larger mass complexes after insulin injection—e.g., alpha, gamma and C subunits (Fig. 3A). In contrast, inhibition of mitochondrial AKT signaling in TAM-treated CAMDAKT mice dramatically decreased the presence of ATP synthase subunits in larger complexes. Moreover, insulin stimulation could not produce formation of the larger molecular mass complexes seen in the control mice.

Co-immunoprecipitation of soluble mitochondrial proteins with AKT1 revealed association with ATP synthase subunits in control mice. However, in TAM-CAMDAKT, this association was significantly reduced (Fig. 3B). Notably, subunits b1 and c, required for proper ATP synthase assembly, were not associated with AKT1 in the TAM-CAMDAKT heart.

Under electron microscopy, sections from CO-CAMDAKT showed mitochondria with distinct and orderly cristae while mitochondria of TAM-CAMDAKT, 7 days after induction, exhibited loss of cristae. Overall mitochondria size was also reduced in TAM-CAMDAKT myocardium. Sarcomere and M line width were lengthened while overall myofibril content was decreased (All p < 0.0001) (Fig. 3C). The lengthening of the sarcomere and reduced density of myofibrils suggest that cardiac contractile strength was reduced, consistent decreased myocardial function.

### Activation of myocardial mitochondrial AKT1 signaling protected against the development of diabetic cardiomyopathy

**Fig. 4:**
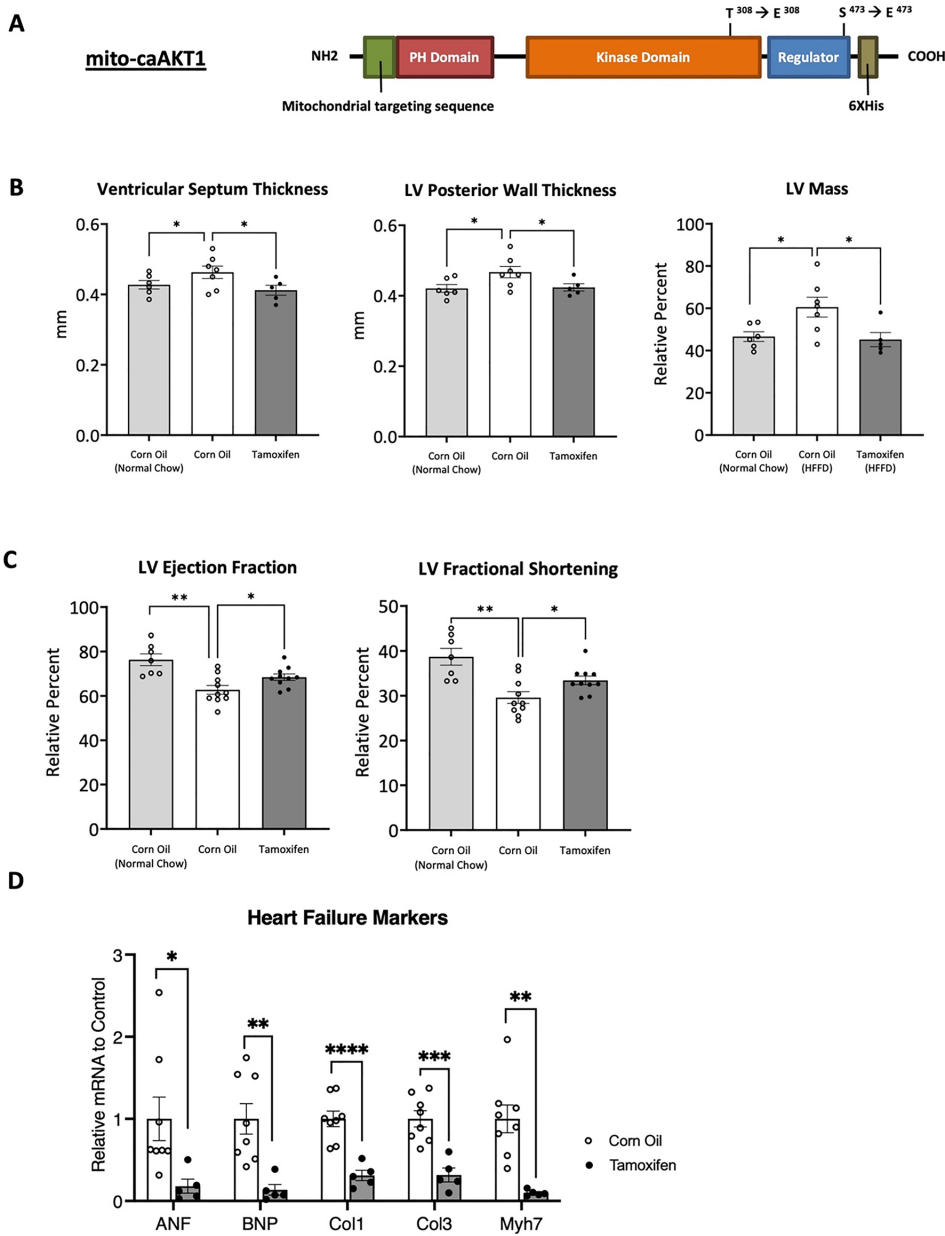

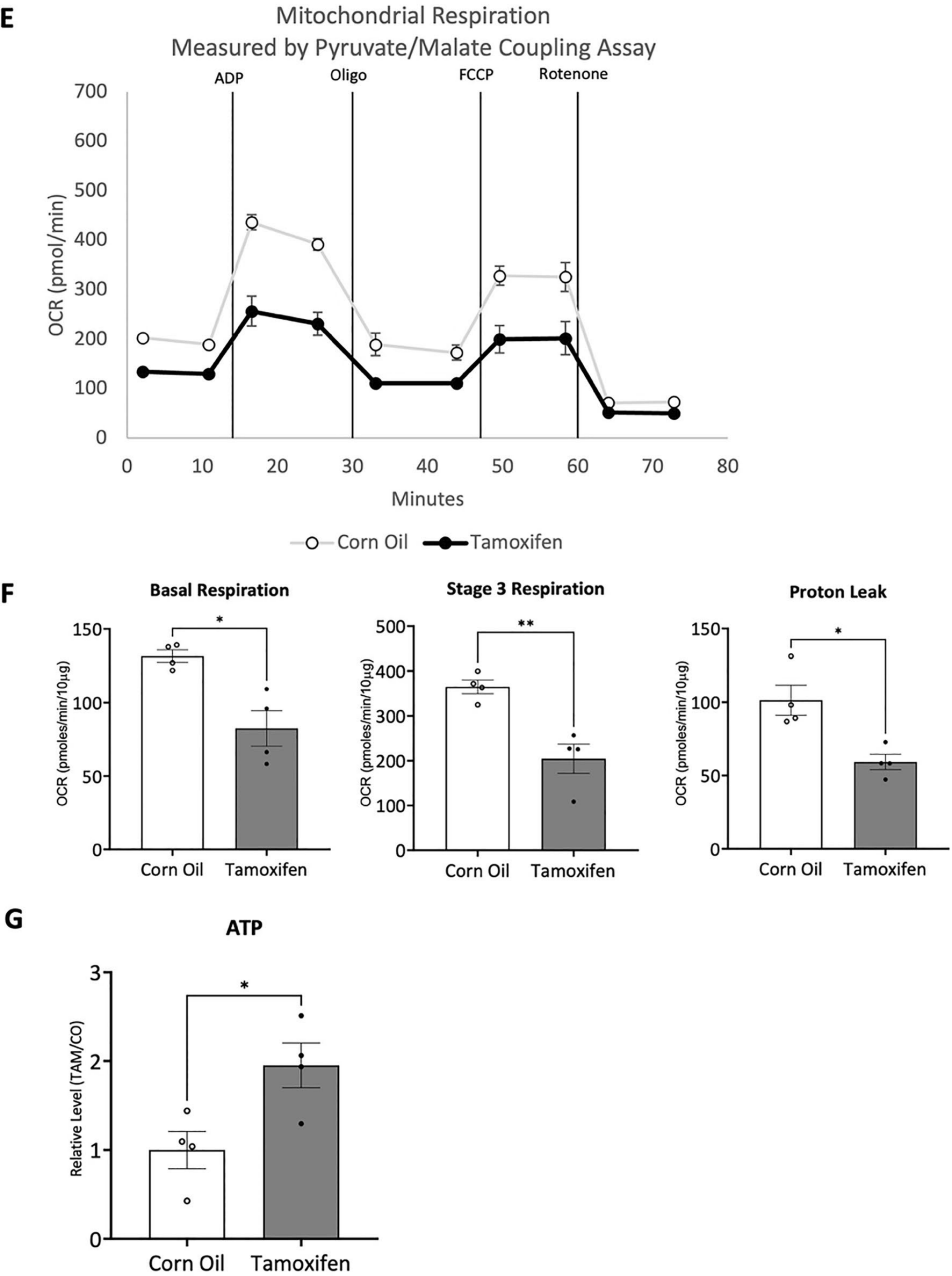
Activation of cardiac mitochondrial AKT1 signaling protected against the development of diabetic cardiomyopathy. **A** The *mito-caAKT1* construct containing a constitutively active AKT1 with a mitochondria localization sequence and 6x-His-tag. Phosphomimetic mutations were made at T308E and S473E. **B** Cardiac left ventricular septum thickness, posterior wall thickness and ventricular mass were analyzed by echocardiogram after 2 months of high fat chow and fructose water diet (HFFD) or normal chow diet, post TAM or CO injection. LV Mass is given as a relative percent of total heart mass (n = 6 (CO – normal chow), n = 7 (CO), n = 5(TAM); All p < 0.05). **C** Cardiac function was measured by echocardiogram after 5 months of HFFD or normal chow post TAM or CO injection. Left ventricular fractional shortening and ejection fraction were maintained in TAM-CAMCAKT mice (n = 7 (CO – normal chow), n = 10 (CO), n = 10 (TAM); All p < 0.05). **D** Expression of markers of heart failure: ANF, BNP, Col1, Col3, My7 measured by RT-qPCR after 5 months of HFFD post TAM or CO injection. Data are presented as relative fold change to CO-CAMCAKT (n = 8 (CO), n = 5(TAM); p < 0.05, < 0.01, < 0.0001, < 0.001, < 0.01). **E** Representative graph of mitochondrial respiration in CAMCAKT cardiac mitochondria after 2 months of HFFD post TAM or CO injection. **F** Basal respiration, stage 3 respiration, and proton leak in CAMCAKT mitochondria were calculated (n = 4; p < 0.05, < 0.01, < 0.05). **G** ATP levels in CAMCAKT mitochondria after 2 months of HFFD post TAM or CO injection. Data are presented as relative levels to CO-CAMCAKT (n = 4, p < 0.05)

If impaired mitochondrial AKT1 is an underlying cause of cardiomyopathy, we hypothesized that, by maintaining its regulatory activity while in a diabetic state, we could attenuate or even prevent the development of diabetic cardiomyopathy. To this end, we generated a bi-transgenic mouse model of constitutively active, cardiac-specific, mitochondria-targeting AKT1 (CAMCAKT). The same transgenic approach was used as in our CAMDAKT model (Fig. 1A, B), but in contrast, the expressed mito-caAKT1 contains phosphomimetic mutations T308E and S473E (Fig. 4A), resulting in a constitutively active mitochondrial AKT1. Male mice were used in the following experiments. CAMCAKT mice were injected with TAM at 8 weeks of age to induce expression of mito-caAKT1, CO as a vehicle control (Additional file 1: Fig. S4A). Expression of mito-caAKT1 was confirmed to be limited to the heart upon TAM injection (Additional file 1: Fig. S4B). Co-localization of mito-caAKT1 to mitochondria was observed by immunofluorescence microscopy (Additional file 1: Fig. S4C). To model the development of diabetes, CAMCAKT mice were placed on a high fat chow and fructose water diet (HFFD) for a period of 2 to 5 months, as specified, after TAM or CO injection at 8 weeks old. Fasting blood glucose levels and body weights of mice fed with HFFD were elevated compared to normal chow fed mice. There were no significant differences between CO-TAMCAKT and TAM-CAMCAKT mice on HFFD (Table 1).

**Table 1:** CAMCAKT mice weights and fasting glucose

	4 Months old/2 months HFFD		7 Months old/5 months HFFD	
	Body weight (g)	Fasting glucose (mg/dl)	Body weight (g)	Fasting glucose (mg/dl)
CO-CAMCAKT	33.5 ± 4.7	201 ± 41	46.6 ± 3.4	225 ± 44
TAM-CAMCAKT	29.9 ± 5.8	185 ± 34	50.2 ± 7.3	230 ± 30
*p-*value	0.12	0.34	0.13	0.75

	4 Months old/normal chow		7 Months old/normal chow	
CO-CAMCAKT	24.2 ± 3.3	145 ± 26	30.6 ± 3.3	180 ± 36

Weights and blood glucose levels, measured after 6 h of fasting, were not significantly altered in the TAM-CAMCAKT compared to CO-CAMCAKT. (n = 12 (HFFD), n = 7 (normal chow))

Heart dimensions and function were measured by echocardiography. After 2 months on HFFD, CO-CAMCAKT mice had measurably larger left ventricle mass, an early pathology of diabetic cardiomyopathy. In comparison, TAM-CAMCAKT hearts had 9% lower posterior wall thickness, and 11% lower ventricle septum thickness. Left ventricle mass was calculated to be 25% lower in TAM-CAMCAKT mice compared to controls (All p < 0.05) (Fig. 4B, Additional file 1: S4D). Ejection fraction and fractional shortening were unchanged (Additional file 1: Fig. S4E). After 5 months on HFFD, reflecting further development of diabetic cardiomyopathy, cardiac function of CO-CAMCAKT mice declined. Ejection fraction in TAM-CAMCAKT mice was 9% higher while fractional shortening was 13% higher (All p < 0.05) (Fig. 4C). Together these results showed that enhancing mitochondrial AKT1 signaling improved cardiac function in a diabetic state.

RNA expression of heart failure markers was quantified by RT-qPCR. Although fibrosis had not yet appeared histologically from the HFFD (Additional file 1: Fig. S4F), Collagen 1 and Collagen 3 expression were significantly downregulated in TAM-CAMCAKT mice. Additional heart failure markers: ANF, BNP, and Myh7 were also found to be significantly lowered in TAM-CAMCAKT mice compared to controls after 5 months of HFFD (p < 0.05, < 0.01, < 0.0001, < 0.001, < 0.01) (Fig. 4D).

After 2 months of HFFD, mitochondria oxygen flux assays were performed to assess mitochondrial function. Mitochondria from TAM-CAMCAKT mice exhibited lower oxygen consumption rates for all induced mitochondrial states, compared to controls (Fig. 4E). TAM-CAMCAKT mitochondrial basal respiration was 37% lower than controls and stage 3 respiration was 44% lower than controls (p < 0.05, < 0.01). Proton leak was 42% lower in TAM-CAMCAKT mitochondria (p < 0.05) (Fig. 4F). Despite lowered oxygen consumption, ATP concentration in TAM-CAMCAKT mitochondria was nearly doubled (p < 0.05) (Fig. 4G). These measurements suggest that constitutively active mito-AKT1 promoted more efficiently coupled respiration.

### Activation of myocardial mitochondrial AKT1 signaling modulated whole-body metabolism in diabetes

**Fig. 5:**
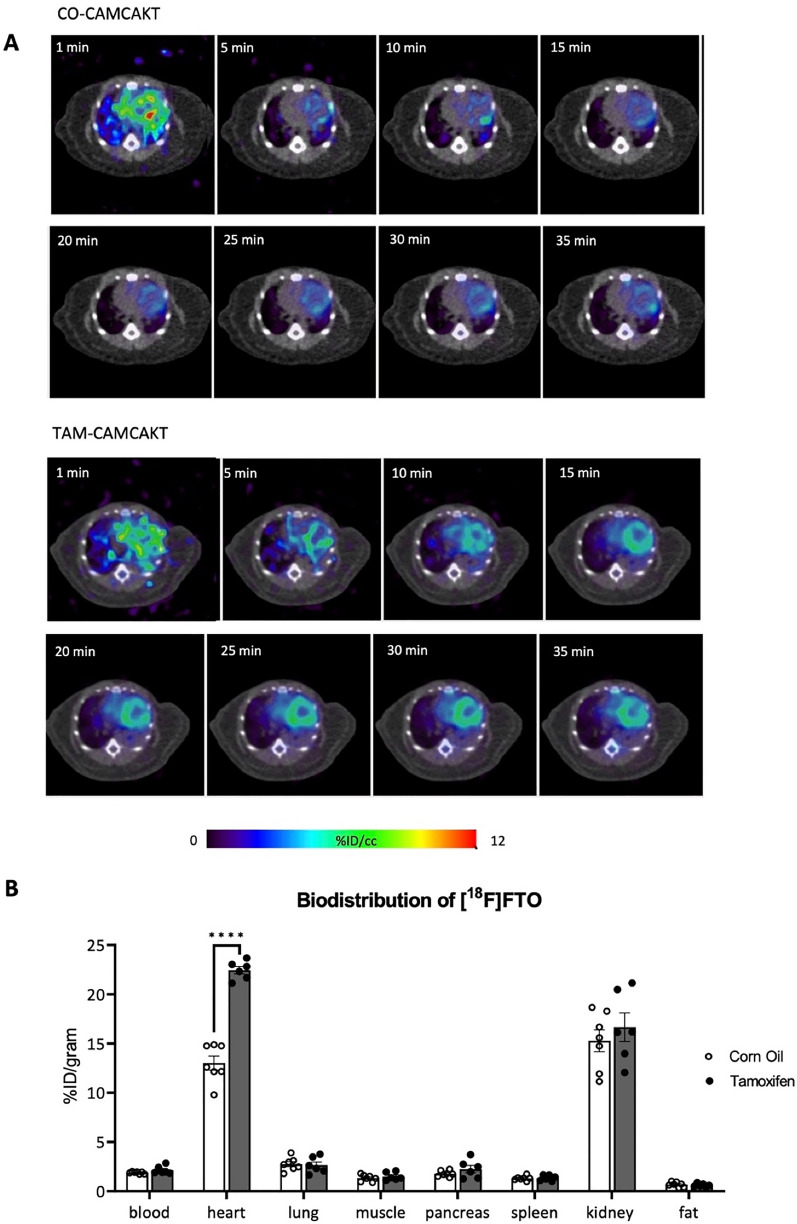

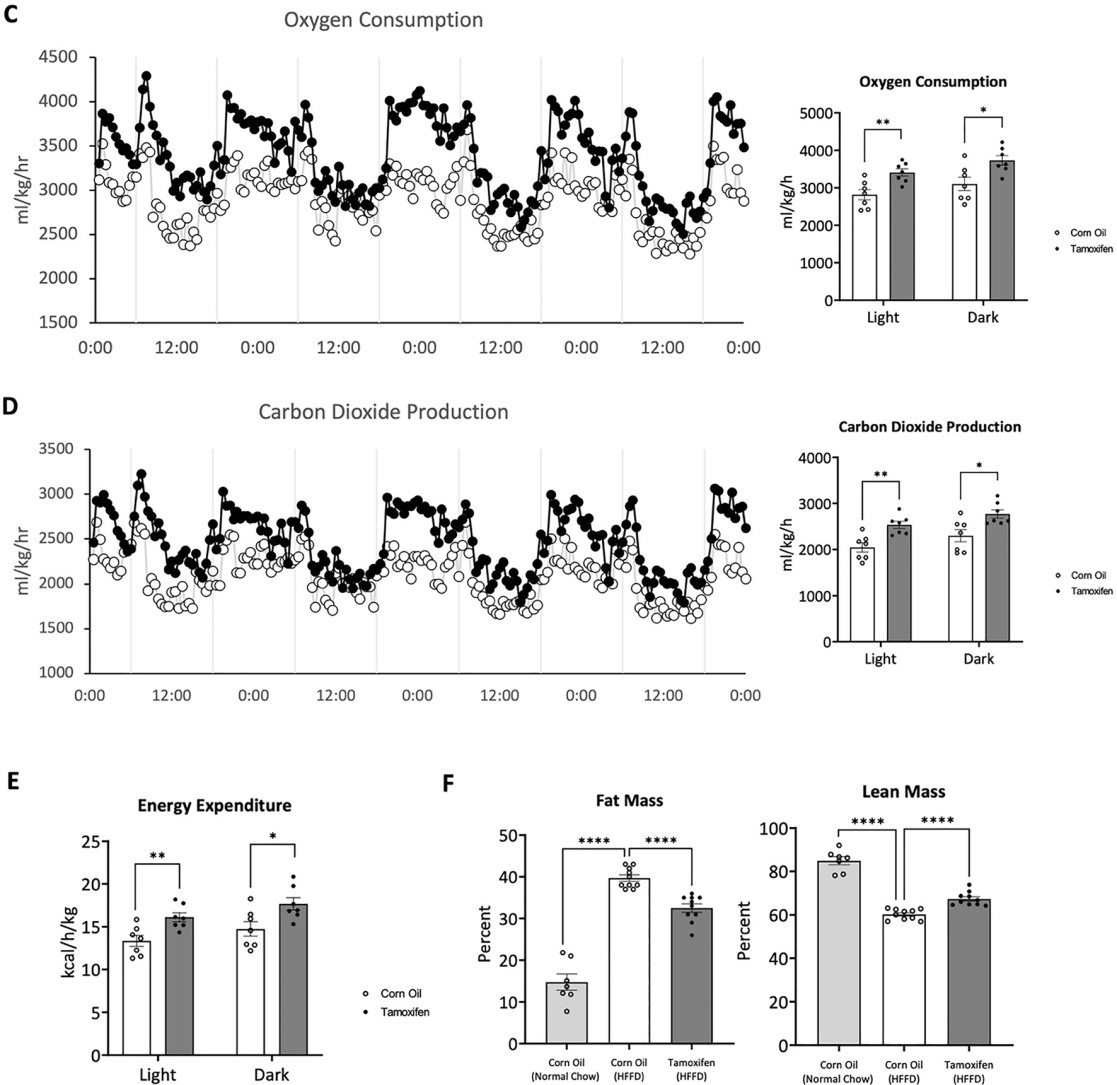

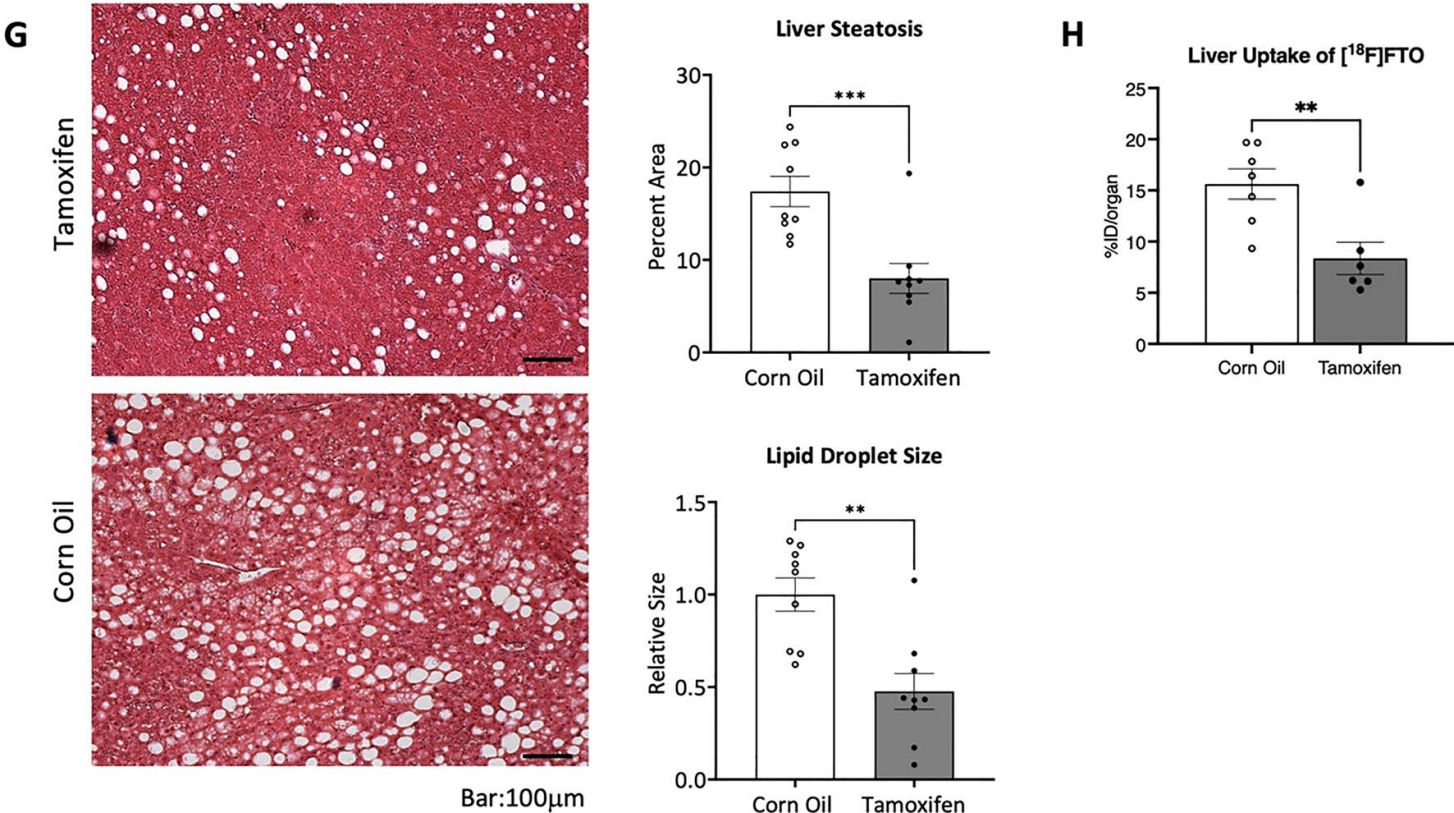
Activation of myocardial mitochondrial AKT1 improved whole body metabolism in diabetes. **A** Representative transverse PET images of differential heart muscle uptake/retention of [^18^F] Fluoro-4-Thia-Oleate (FTO). CAMCAKT mice placed on 2 months of HFFD after TAM or CO injection were administered 7.4 MBq [^18^F]FTO followed by a 35 min dynamic scan. **B** Organ biodistribution (%ID/gram) of [^18^F]FTO in CAMCAKT after 2 months of HFFD following TAM or CO injection. Mice were administered 7.4 MBq [^18^F]FTO followed by tissue collection after 40 min (n = 7 (CO), n = 6 (TAM); p < 0.0001). **C** Metabolic cage analysis was performed on CAMCAKT mice after 2 months of HFFD following TAM or CO injection. Kinetic data for oxygen consumption (n = 7 each group, p < 0.01, < 0.05) and **D** carbon dioxide production are shown as the mean for each time point and summarized as means for light and dark periods (n = 7 each group, p < 0.01, < 0.05). E. Energy expenditure was calculated from metabolic cage measurements (n = 7 each group, p < 0.01, < 0.05). **F** Body composition measurements of CAMCAKT after 2 months of HFFD following TAM or CO injection (n = 7 (CO–normal chow), n = 10 (CO), n = 10 (TAM); p < 0.0001). **G** Left, representative images of H&E-stained liver sections showed reduced liver steatosis in TAM-CAMCAKT mice after 2 months of HFFD. Right, lipid droplet percent area and size were quantified. Scale bar = 100 μm (n = 9 each group; p < 0.001, 0.01). **H** Liver uptake (%ID/organ) of [^18^F]FTO in CAMCAKT mice after 2 months of HFFD following TAM or CO injection. Mice were administered 7.4 MBq [^18^F]FTO followed by tissue collection after 40 min (n = 7 (CO), n = 6 (TAM); p < 0.01)

Fatty acids are the major source of energy for the heart and their metabolism within the mitochondria is critical for maintaining cardiac function. Here, positron emission tomography (PET), a non-invasive high sensitivity imaging modality, was used to monitor the target radiolabeled [^18^F]fluoro-4-thia-oleate (FTO) probe in various tissues in vivo. This third-generation fatty acid probe, an oleic acid derivative, was chosen for its high cardiac uptake and longer retention compared to alternative fatty acid probes, making it invaluable for measuring the modulation of fatty acid utilization in our transgenic mice [29]. CAMCAKT mice were administered with 7.4 MBq [^18^F]FTO after 2 months of HFFD following CO or TAM injection. The uptake of [^18^F]FTO in both groups of mice were compared by microPET imaging followed by terminal ex vivo biodistribution analyses. The representative dynamic microPET images from 0 to 35 min in regions of interest were shown (Fig. 5A). As expected, microPET imaging provided clear visualizations of the cardiac accumulation of [^18^F]FTO in TAM-CAMCAKT mice. The result of tissue biodistribution confirmed that cardiac-specific uptake value in TAM-CAMCAKT mice were 42% higher than the uptake value of CO-CAMCAKT mice (p < 0.0001) (Fig. 5B). Of note, fat deposition was very low in CO-CAMCAKT and TAM-CAMCAKT mice, and the difference was insignificant between these two groups (Additional file 1: Fig. S5A).

Next, utilizing metabolic cages to measure whole body metabolism, mice were individually housed and monitored to track oxygen consumption and carbon dioxide production after 2 months of HFFD following TAM or CO injection. Male TAM-CAMCAKT mice consumed 20% and 21% more oxygen than CO-CAMCAKT during the light and dark periods respectively, normalized to body weight (p < 0.05) (Fig. 5C). The same mice produced 21% and 24% more carbon dioxide than controls during light and dark periods respectively (p < 0.05) (Fig. 5D). Male TAM-CAMCAKT mice expended 20% and 21% more energy, normalized to body weight, compared to controls during the day and night periods (p < 0.05) (Fig. 5E). Food intake was unchanged between the two groups. The respiratory exchange ratio was unchanged (Additional file 1: Fig. S5A). Basal levels of serum free fatty acid were similar between TAM (0.40 $\pm$ 0.023 nmol/μl) and CO-CAMCAKT after 2 months of HFFD (0.48 $\pm$ 0.065 nmol/μl) (p = 0.30). Glucose tolerance was also unchanged between the CO and TAM-CAMCAKT after two months of HFFD (Additional file 1: Fig. S5B).

Body composition was measured using an NMR-MRI based body analyzer. At 8 weeks old, CAMCAKT mice were induced. All mice started with a mean of 10% total body fat. After 2 months of HFFD, TAM-CAMCAKT mice had 18% less body fat relative to CO-CAMCAKT and 12% greater lean mass (All p < 0.0001) (Fig. 5F). After 5 months of HFFD, both groups reached a plateau of 40% total body fat.

Fatty liver was markedly reduced in TAM-CAMCAKT mice. Liver steatosis was quantified by image analysis of H&E-stained sections. Liver steatosis was measured after multiple durations on HFFD and as expected, lipid accumulation became progressively more severe. CAMCAKT mice at 8 weeks old began with 5% steatosis by percent area of lipid droplet. After induction, mice were placed on HFFD. After 2 months, the area of lipid droplets was 54% less in TAM-CAMCAKT mice, and the average droplet size 52% smaller (p < 0.001, 0.01) (Fig. 5G). Consistent with those findings, hepatic uptake of [^18^F]FTO was 46% lower in TAM-CAMCAKT mice after 2 months of HFFD (p < 0.01) (Fig. 5H). Livers from TAM-CAMCAKT mice consistently had less lipid accumulation up to 5 months of HFFD, at which point there was still a 22% reduction of steatosis by area (p < 0.05) (Additional file 1: Fig. S5B). These results showed that activation of cardiac mitochondrial AKT1 improved whole body metabolism and had a pronounced effect on the liver.

## Discussion

### Inhibition of mitochondrial AKT1 and cardiomyopathy

CAMDAKT mice served as a model of the effects of metabolic syndrome as it pertains to mitochondrial AKT1 signaling and displayed a severe cardiomyopathic phenotype. Cardiac fibrosis and a decline of left ventricle function developed quickly after the induction of dominant negative mito-AKT1. Due to the severe phenotype, we did not further induce metabolic syndrome in CAMDAKT mice. A HFFD on top of the loss of function design could make data interpretation difficult and deviate from our intended hypothesis testing effort. Additionally, long term survival rates of CAMDAKT mice were poor due to the overexpression of dominant negative mitochondrial AKT1.

Looking deeper, we found disruption of mitochondrial cristae structure, reduced mitochondrial respiration efficiency, and malformation of the ATP synthase complex in our model. Proper assembly of ATP synthase requires the joining of the F_0_-inner mitochondrial membrane-spanning subcomplex to the F_1_ soluble subcomplex extending into the mitochondrial matrix [30]. Together, F_1_/F_0_ form a nucleotide binding site and is the catalytic site for ATP production. In addition to ATP production, ATP synthase complexes are organized into dimers oriented at 90° and reinforce the cristae [31]. Cristae in mitochondria greatly increase the surface area available to carry out electron transfer, maintain electrochemical gradient, and facilitate ATP production, hence why mitochondria become dysfunctional when this structure is lost [32, 33]. Tight packing of cristae is characteristic and necessary in cardiac mitochondria to facilitate a higher flux of molecules across the membrane [31]. Loss of mitochondrial AKT1 signaling compromised these critical facets of mitochondrial structure and function.

The data reported in this study demonstrated the effects of AKT1 signaling on mitochondrial structure and respiratory function. Our previous studies have shown that mitochondrial AKT1 interacted with oxidative phosphorylation complexes, modulated mitochondrial calcium influx, and decreased oxidative stress [15]. Oxidative phosphorylation, electron transport, calcium flux, and oxidative stress in mitochondria are interconnected processes that influence each other's activity and have significant implications for mitochondrial function. The precise balance among these processes is crucial. These new findings provide new evidence that mitochondrial AKT1 signaling is involved in multiple steps of mitochondrial metabolism.

### Activation of mitochondrial AKT1 attenuated diabetic cardiomyopathy

Augmenting myocardial mitochondrial AKT1 in CAMCAKT mice attenuated the development of DCM. We successfully produced a phenotype of DCM through high fat and fructose diet. Mice presented enlarged left ventricles after 2 months of HFFD, characteristic of early-stage cardiomyopathy. After 5 months, heart function was diminished, a later stage consequence of the disease [34]. In early and later time points, we confirmed that enhancing mitochondrial AKT1 signaling was able to reduce hypertrophy and improve cardiac function respectively.

Mitochondrial dysfunction is a key contributing factor to cardiomyopathy. Both human and animal studies have demonstrated elevated myocardial oxygen consumption and lowered cardiac respiration efficiency in diabetic hearts [5]. Mitochondrial uncoupling and proton leak could have increased oxygen consumption, while simultaneously reducing ATP production [35]. A basal level of uncoupling is necessary for heat generation and regulatory functions, but uncoupling is significantly elevated in insulin deficient conditions [35]. In our model, mitochondria with augmented AKT1 signaling isolated from myocardium consumed less oxygen in basal and stage 3 respiration while ATP content was notably higher. These findings indicate that it is possible to improve the efficiency of myocardial respiration through activation of mitochondrial AKT1 signaling.

Markers of heart failure were significantly reduced by mitochondrial AKT1 signaling. ANF and BNP are produced by myocytes in the atria and ventricles, respectively, in response to myocardial strain due to increased pressure or an overload in output demand [36]. Elevated BNP is widely accepted as the gold standard biomarker for diagnosing heart failure [37]. Myosin heavy chain 7 (Myh7) is a newly characterized marker for heart failure; in vivo overexpression studies of Myh7 resulted in cardiac dilation, and cardiac failure [38]. These results further corroborate the effects of mitochondrial AKT1 on improving DCM.

### Myocardial mitochondrial AKT1 effects on whole body metabolism

Interestingly, improving cardiac function in DCM had metabolic benefits that extended beyond the heart. Augmenting mitochondrial AKT1 in CAMCAKT mice resulted in a leaner body composition after 2 months of HFFD. This difference diminished over time as all mice reached a plateau of adiposity by 5 months of HFFD, suggesting that a continuous high calorie diet eventually obfuscated changes brought about by cardiac mitochondrial AKT1 signaling. Considering that we only directly modulated cardiac signaling, our data suggests that while the heart maintained optimal function throughout the study period, its metabolic contribution became proportionally reduced as weight gain and fat accumulation increased.

Our CAMCAKT model showed that mitochondrial AKT1 signaling promoted higher whole body energy expenditure. This manifested in higher oxygen consumption, but isolated mitochondria from these mice showed lower oxygen consumption. It can be problematic to compare these two methods directly as the Seahorse extracellular flux assay measured the maximal potential respiration of a normalized quantity of isolated mitochondria under substrate saturating conditions, while the metabolic cage measured the sum of energy expenditure of a live mouse from all tissues and organs. Taken together, our data suggested that CAMCAKT mice had higher overall energy expenditure while simultaneously, at the mitochondrial level, metabolized energy substrates with more tightly coupled and efficient respiration in cardiac muscle.

While the heart is metabolically flexible, fatty acids are the primary fuel source to meet its high energy demands [39]. Cardiac mitochondrial AKT1 signaling promoted fatty acid metabolism under HFFD, leading to higher whole body energy expenditure and reduced body fat. Reduced adiposity has potential metabolic and cardiovascular benefits [40]. Accompanying increased metabolism, fatty liver induced by HFFD was markedly attenuated in our model of augmented mitochondrial AKT1. Elevated fatty acid influx and increased fatty acid esterification resulting in intrahepatic triglyceride accumulation contribute to the development of fatty liver [41]. In our HFFD model, increased cardiac fatty acid update in TAM-CAMCAKT mice could have reduced hepatic fatty acid flux and fatty acid uptake as shown in the PET scan, thereby attenuating the development of fatty liver. We consistently saw reduced hepatic steatosis up to five months on the HFFD. This occurred despite the fact that differences in whole body adiposity narrowed as the duration of the HFFD increased. These findings indicated intermodulation of metabolism in the heart and liver, which warrants further investigation.

There were some limitations in our current study. We used male mice for the majority of the experiments in this project, with selective experiments on myocardial phenotype confirmed in female mice. The effects of biological sex were not fully explored in this study. Additionally, we focused on a 2-month HFFD study design to interrogate the metabolic changes in the body. Future studies are needed to fully explore the changes of fatty acid metabolism and further elucidate of the mechanism by which mitochondrial AKT1 modulates changes in whole body metabolism.

## Conclusions

A large body of evidence indicates that mitochondria are a key factor in maintaining normal cardiac muscle function and that mitochondrial dysfunction is involved in the development of cardiomyopathy. Extensive data from human studies and experimental models suggest that insulin resistance is implicated in the development of DCM [42–44]. How altered insulin receptor signaling, in the setting of insulin resistance, contributes to the development of mitochondrial dysfunction and thereby precipitates development of heart failure remains to be elucidated. Our previous work established the role of AKT1 as a signaling mediator between insulin receptors and cardiac mitochondria. The present study showed that in vivo impairment of cardiac mitochondrial AKT1 signaling led to acute cardiomyopathy while augmenting this signaling protected the heart against the development of DCM, as well as improved whole-body metabolism.

Discoveries in molecular signaling and metabolic biomarkers pose new opportunities to challenge the notion that metabolic cardiomyopathy is a progressive disease and ultimately leads to poor clinical outcomes in the presence of co-morbidities. The mechanisms of myocardial energy dysregulation and associated adaptive biochemical events are complex during development of heart failure, with multiple pathways converging on mitochondria [39]. Although mitochondrial dysfunction has been implicated in the development of various cardiomyopathies, effective intervention to prevent or reverse mitochondrial dysfunction is not yet available. Further understanding the mechanisms of mitochondrial AKT1 signaling and its regulation of ATP synthase may create new opportunities to develop novel strategies to improve mitochondrial function and reverse the progressive decline of myocardial function and regain healthier whole-body metabolism.

### Supplementary Information

**Additional file 1: Figure S1.** Cardiac-specific induction of Cre recombinase after TAM Induction in CAMDAKT Mice. A. CAMDAKT mice were administered TAM or CO once, as indicated at 2 months of age. 10 h after tamoxifen injection, the presence of Cre recombinase in the nuclei was examined by immunohistochemistry analysis. **Figure S2.** Development of Myocardial Fibrosis and Cardiomyopathy Caused by Impaired Mitochondrial AKT1 Signaling. A. Representative images of trichrome stained mid-heart cross sections. TAM or CO was administered once, as indicated. 7 days after injection, cardiac fibrosis was quantified by trichrome staining. Scale bar = 500 μm. B. Hearts were weighed and reported as a ratio of heart mass to body mass. (n = 6 (TAM-Myh6-Cre), n = 6 (TAM-MDNAKT), n = 5 (CO-CAMDAKT), n = 13 (TAM-CAMDAKT); p < 0.05). C. Left, representative images of trichrome stained myocardial sections. TAM or CO was administered once, as indicated. 7 days after injection, cardiac fibrosis was quantified by trichrome staining. Scale bar = 100 μm. Right, quantification of cardiac fibrosis. (n = 10 (TAM-Myh6-Cre), n = 9 (TAM-MDNAKT), n = 9 (CO-CAMDAKT), n = 9 (TAM-CAMDAKT); p < 0.01). **Figure S3.** AKT1 Interacted with the ATP Synthase Complex. A. Scheme of sucrose gradient used to separate mitochondrial protein complexes. **Figure S4.** Generation of Transgenic Mice with Inducible Cardiomyocyte-Specific Expression of Mitochondria-Targeting Constitutively Active AKT1. A. Scheme of model to overexpress cardiac-specific mitochondria-targeting constitutively active AKT1 (CAMCAKT). Transgenic mice harboring *mito-caAkt1* were crossed with Myh6-Cre mice (Cre recombinase expressed in cardiomyocytes) to generate CAMCAKT bi-genic mice for this series of experiments. The Neo cassette, containing Neo cDNA followed by an SV40 PolyA signal to terminate transcription, is removed by Cre-mediated recombination upon injection with tamoxifen (TAM). B. Cardiac specific expression in CAMCAKT mice after TAM induction. Total protein was isolated from the specified organs of CAMCAKT mice 10 h after TAM injection. Tamoxifen-induced mito-caAKT1 protein expression was detected by 6x-His antibodies in the heart specifically and not detected in other organs examined. Actinin was used as a loading control. C. Mitochondria specific localization of mito-caAKT1. Co-localization of mito-caAKT1 and mitochondria was visualized by immunostaining. 24 h after injection with TAM, hearts were fixed and embedded with paraffin. 4-micron thin sections were immunostained with 6x-His antibodies (red) followed by staining with MitoTracker™ Green FM (green) and DAPI (blue). Images were taken within 6 h after staining was finished. Mito-caAKT1was detected in TAM-CAMCAKT cardiomyocytes and colocalized with mitochondria. Scale bar = 50 μm (upper), 20 μm (lower). D. Representative echocardiograms in the parasternal long-axis view, showing measurements of the left ventricle. E. Cardiac function was measured by echocardiogram after 2 months of HFFD following TAM or CO injection (n = 6 (CO–normal chow), n = 7 (CO), n = 5 (TAM)). F. Representative images of trichrome stained CAMCAKT myocardial sections. Cardiac fibrosis was not detected by trichrome staining. Scale bar = 100 μm. **Figure S5.** Mitochondrial AKT1 Improved Metabolism in Diet-Induced Diabetes. A. Representative images of Oil Red O stained CAMCAKT myocardial sections after 2 months of HFFD following TAM or CO injection. (n = 5 each group) Scale bar = 50 μm. B. Oral glucose tolerance. Mice were given 2 g/kg of glucose after a 5 h fast. Blood glucose levels were unchanged between CO and TAM-CAMCAKT after 2 months of HFFD (n = 4 (CO–normal chow), n = 5 (CO), n = 4 (TAM). C. Respiratory exchange ratio, summarized as means for light and dark periods, were unchanged (n = 7 each group). D. Liver steatosis was reduced in TAM-CAMCAKT mice up to 5 months on HFFD (n = 8 each group; p < 0.05).

**Additional file 2.** Further information of the experimental methods.

## Data Availability

The datasets used and/or analyzed during the current study are available from the corresponding author on reasonable request.

## Abbreviations

CAMDAKT: Transgenic mouse with inducible expression of cardiac-specific, mitochondria-targeting dominant negative AKT1
CAMCAKT: Transgenic mouse with inducible expression of cardiac-specific, mitochondria-targeting constitutively active AKT1
CO: Corn oil
DCM: Diabetic cardiomyopathy
FTO: Fluoro-4-thia-oleate
LV: Left Ventricle
MDNAKT: Transgenic mouse harboring mito-dnAKT construct
mito-dnAkt: Mitochondria targeting dominant-negative Atk1 construct
Myh6-Cre: Transgenic mouse with Myh6 promoter driven inducible Cre recombinase
PKB: Protein kinase B
TAM: Tamoxifen
WT: Wild type

## References

[1] Bugger H, Abel ED (2008). Molecular mechanisms for myocardial mitochondrial dysfunction in the metabolic syndrome. Clin Sci (Lond).

[2] Scheuermann-Freestone M, Madsen PL, Manners D, Blamire AM, Buckingham RE, Styles P (2003). Abnormal cardiac and skeletal muscle energy metabolism in patients with type 2 diabetes. Circulation.

[3] Ye G, Metreveli NS, Donthi RV, Xia S, Xu M, Carlson EC (2004). Catalase protects cardiomyocyte function in models of type 1 and type 2 diabetes. Diabetes.

[4] Pierce GN, Dhalla NS (1985). Heart mitochondrial function in chronic experimental diabetes in rats. Can J Cardiol.

[5] Boudina S, Sena S, O'Neill BT, Tathireddy P, Young ME, Abel ED (2005). Reduced mitochondrial oxidative capacity and increased mitochondrial uncoupling impair myocardial energetics in obesity. Circulation.

[6] Savabi F (1988). Mitochondrial creatine phosphokinase deficiency in diabetic rat heart. Biochem Biophys Res Commun.

[7] Veksler VI, Murat I, Ventura-Clapier R (1991). Creatine kinase and mechanical and mitochondrial functions in hereditary and diabetic cardiomyopathies. Can J Physiol Pharmacol.

[8] Taniguchi CM, Emanuelli B, Kahn CR (2006). Critical nodes in signalling pathways: insights into insulin action. Nat Rev Mol Cell Biol.

[9] White MF (2006). Regulating insulin signaling and beta-cell function through IRS proteins. Can J Physiol Pharmacol.

[10] Sale EM, Sale GJ (2008). Protein kinase B: signalling roles and therapeutic targeting. Cell Mol Life Sci.

[11] Coffer PJ, Geijsen N, M'Rabet L, Schweizer RC, Maikoe T, Raaijmakers JA (1998). Comparison of the roles of mitogen-activated protein kinase kinase and phosphatidylinositol 3-kinase signal transduction in neutrophil effector function. Biochem J.

[12] Alessi DR, Caudwell FB, Andjelkovic M, Hemmings BA, Cohen P (1996). Molecular basis for the substrate specificity of protein kinase B; comparison with MAPKAP kinase-1 and p70 S6 kinase. FEBS Lett.

[13] Yang JY, Deng W, Chen Y, Fan W, Baldwin KM, Jope RS (2013). Impaired translocation and activation of mitochondrial Akt1 mitigated mitochondrial oxidative phosphorylation Complex V activity in diabetic myocardium. J Mol Cell Cardiol.

[14] Yang JY, Yeh HY, Lin K, Wang PH (2009). Insulin stimulates Akt translocation to mitochondria: implications on dysregulation of mitochondrial oxidative phosphorylation in diabetic myocardium. J Mol Cell Cardiol.

[15] Deng W, Leu HB, Chen Y, Chen YH, Epperson CM, Juang C (2014). Protein kinase B (PKB/AKT1) formed signaling complexes with mitochondrial proteins and prevented glycolytic energy dysfunction in cultured cardiomyocytes during ischemia-reperfusion injury. Endocrinology.

[16] Su CC, Yang JY, Leu HB, Chen Y, Wang PH (2012). Mitochondrial Akt-regulated mitochondrial apoptosis signaling in cardiac muscle cells. Am J Physiol Heart Circ Physiol.

[17] Gilbert RE (2017). Proximal tubulopathy: prime mover and key therapeutic target in diabetic kidney disease. Diabetes.

[18] Matsuda T, Cepko CL (2007). Controlled expression of transgenes introduced by in vivo electroporation. Proc Natl Acad Sci U S A.

[19] Kanegae Y, Lee G, Sato Y, Tanaka M, Nakai M, Sakaki T (1995). Efficient gene activation in mammalian cells by using recombinant adenovirus expressing site-specific Cre recombinase. Nucleic Acids Res.

[20] Soriano P (1999). Generalized lacZ expression with the ROSA26 Cre reporter strain. Nat Genet.

[21] Pettitt SJ, Liang Q, Rairdan XY, Moran JL, Prosser HM, Beier DR (2009). Agouti C57BL/6N embryonic stem cells for mouse genetic resources. Nat Methods.

[22] Lin HY, Chen Y, Chen YH, Ta AP, Lee HC, MacGregor GR (2021). Tubular mitochondrial AKT1 is activated during ischemia reperfusion injury and has a critical role in predisposition to chronic kidney disease. Kidney Int.

[23] Du XJ (2004). Gender modulates cardiac phenotype development in genetically modified mice. Cardiovasc Res.

[24] Wang CY, Liao JK (2012). A mouse model of diet-induced obesity and insulin resistance. Methods Mol Biol.

[25] Casimiro I, Stull ND, Tersey SA, Mirmira RG (2021). Phenotypic sexual dimorphism in response to dietary fat manipulation in C57BL/6J mice. J Diabetes Complications.

[26] Egemnazarov B, Crnkovic S, Nagy BM, Olschewski H, Kwapiszewska G (2018). Right ventricular fibrosis and dysfunction: actual concepts and common misconceptions. Matrix Biol.

[27] Csecs I, Pashakhanloo F, Paskavitz A, Jang J, Al-Otaibi T, Neisius U (2020). Association between left ventricular mechanical deformation and myocardial fibrosis in nonischemic cardiomyopathy. J Am Heart Assoc.

[28] Vercellino I, Sazanov LA (2022). The assembly, regulation and function of the mitochondrial respiratory chain. Nat Rev Mol Cell Biol.

[29] DeGrado TR, Bhattacharyya F, Pandey MK, Belanger AP, Wang S (2010). Synthesis and preliminary evaluation of 18-(18)F-fluoro-4-thia-oleate as a PET probe of fatty acid oxidation. J Nucl Med.

[30] Ruhle T, Leister D (2015). Assembly of F1F0-ATP synthases. Biochim Biophys Acta.

[31] Brandt T, Mourier A, Tain LS, Partridge L, Larsson NG, Kuhlbrandt W (2017). Changes of mitochondrial ultrastructure and function during ageing in mice and Drosophila. Elife.

[32] Bornhovd C, Vogel F, Neupert W, Reichert AS (2006). Mitochondrial membrane potential is dependent on the oligomeric state of F1F0-ATP synthase supracomplexes. J Biol Chem.

[33] Davies KM, Anselmi C, Wittig I, Faraldo-Gomez JD, Kuhlbrandt W (2012). Structure of the yeast F1Fo-ATP synthase dimer and its role in shaping the mitochondrial cristae. Proc Natl Acad Sci U S A.

[34] Jia G, Hill MA, Sowers JR (2018). Diabetic cardiomyopathy: an update of mechanisms contributing to this clinical entity. Circ Res.

[35] Crescenzo R, Bianco F, Mazzoli A, Giacco A, Liverini G, Iossa S (2014). Mitochondrial efficiency and insulin resistance. Front Physiol.

[36] Brandt RR, Wright RS, Redfield MM, Burnett JC (1993). Atrial natriuretic peptide in heart failure. J Am Coll Cardiol.

[37] Gaggin HK, Januzzi JL (2013). Biomarkers and diagnostics in heart failure. Biochim Biophys Acta.

[38] Peter AK, Rossi AC, Buvoli M, Ozeroff CD, Crocini C, Perry AR (2019). Expression of normally repressed myosin heavy chain 7b in the mammalian heart induces dilated cardiomyopathy. J Am Heart Assoc.

[39] Lopaschuk GD, Karwi QG, Tian R, Wende AR, Abel ED (2021). Cardiac energy metabolism in heart failure. Circ Res.

[40] Heymsfield SB, Wadden TA (2017). Mechanisms, pathophysiology, and management of obesity. N Engl J Med.

[41] Loomba R, Friedman SL, Shulman GI (2021). Mechanisms and disease consequences of nonalcoholic fatty liver disease. Cell.

[42] Bugger H, Riehle C, Jaishy B, Wende AR, Tuinei J, Chen D (2012). Genetic loss of insulin receptors worsens cardiac efficiency in diabetes. J Mol Cell Cardiol.

[43] Cook SA, Varela-Carver A, Mongillo M, Kleinert C, Khan MT, Leccisotti L (2010). Abnormal myocardial insulin signalling in type 2 diabetes and left-ventricular dysfunction. Eur Heart J.

[44] Qi Y, Xu Z, Zhu Q, Thomas C, Kumar R, Feng H (2013). Myocardial loss of IRS1 and IRS2 causes heart failure and is controlled by p38alpha MAPK during insulin resistance. Diabetes.

