# Supplementary material for: Dual roles of myocardial mitochondrial AKT on diabetic cardiomyopathy and whole body metabolism
Source: Cardiovasc Diabetol. 2023 Oct 27;22:294. doi: 10.1186/s12933-023-02020-1 (PMC10612246; doi:10.1186/s12933-023-02020-1)
Supplement: Supplementary file 1 — Additional file 1: Figure S1. Cardiac-specific induction of Cre recombinase after TAM Induction in CAMDAKT Mice. A. CAMDAKT mice were administered TAM or CO once, as indicated at 2 months of age. 10 h after tamoxifen injection, the presence of Cre recombinase in the nuclei was examined by immunohistochemistry analysis. Figure S2. Development of Myocardial Fibrosis and Cardiomyopathy Caused by Impaired Mitochondrial AKT1 Signaling. A. Representative images of trichrome stained mid-heart cross sections. TAM or CO was administered once, as indicated. 7 days after injection, cardiac fibrosis was quantified by trichrome staining. Scale bar = 500 μm. B. Hearts were weighed and reported as a ratio of heart mass to body mass. (n = 6 (TAM-Myh6-Cre), n = 6 (TAM-MDNAKT), n = 5 (CO-CAMDAKT), n = 13 (TAM-CAMDAKT); p < 0.05). C. Left, representative images of trichrome stained myocardial sections. TAM or CO was administered once, as indicated. 7 days after injection, cardiac fibrosis was quantified by trichrome staining. Scale bar = 100 μm. Right, quantification of cardiac fibrosis. (n = 10 (TAM-Myh6-Cre), n = 9 (TAM-MDNAKT), n = 9 (CO-CAMDAKT), n = 9 (TAM-CAMDAKT); p < 0.01). Figure S3. AKT1 Interacted with the ATP Synthase Complex. A. Scheme of sucrose gradient used to separate mitochondrial protein complexes. Figure S4. Generation of Transgenic Mice with Inducible Cardiomyocyte-Specific Expression of Mitochondria-Targeting Constitutively Active AKT1. A. Scheme of model to overexpress cardiac-specific mitochondria-targeting constitutively active AKT1 (CAMCAKT). Transgenic mice harboring mito-caAkt1 were crossed with Myh6-Cre mice (Cre recombinase expressed in cardiomyocytes) to generate CAMCAKT bi-genic mice for this series of experiments. The Neo cassette, containing Neo cDNA followed by an SV40 PolyA signal to terminate transcription, is removed by Cre-mediated recombination upon injection with tamoxifen (TAM). B. Cardiac specific expression in CAMCAKT mice after TAM inductio [file 12933_2023_2020_MOESM1_ESM.pptx]

## Slide 1
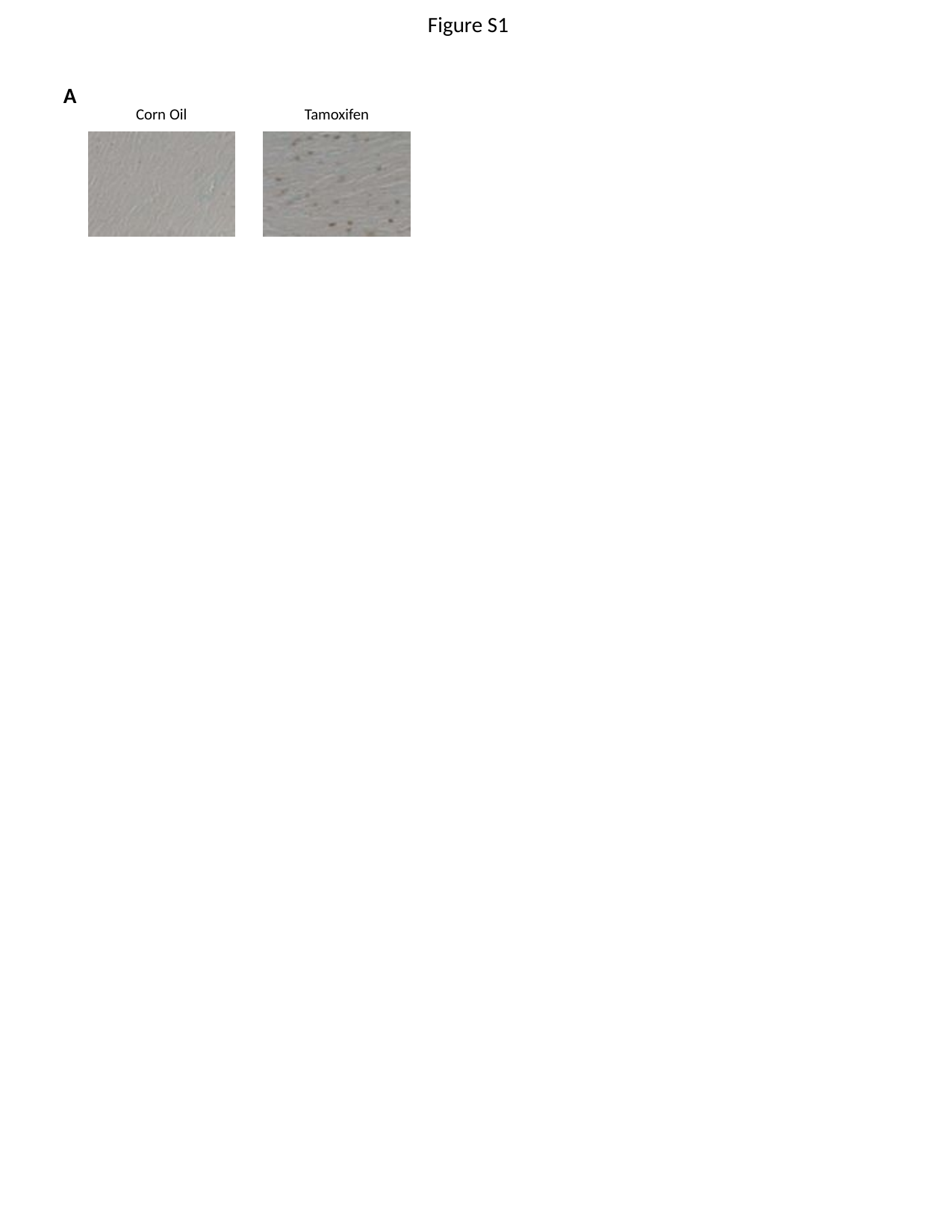

Figure S1
A
Corn Oil
Tamoxifen

## Slide 2
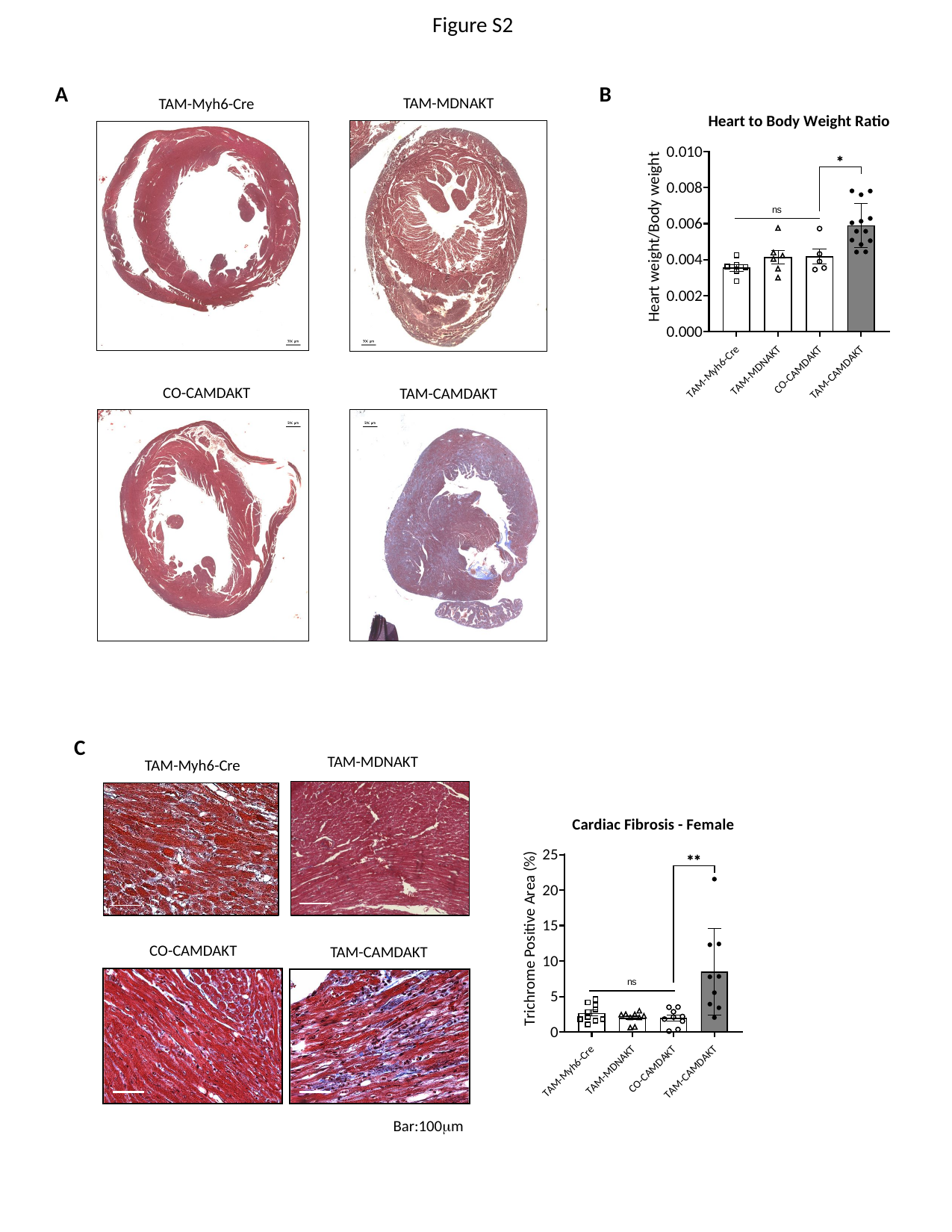

Figure S2
A
B
TAM-MDNAKT
TAM-Myh6-Cre
CO-CAMDAKT
TAM-CAMDAKT
C
Corn Oil
Bar:100μm
TAM-MDNAKT
TAM-Myh6-Cre
CO-CAMDAKT
TAM-CAMDAKT

## Slide 3
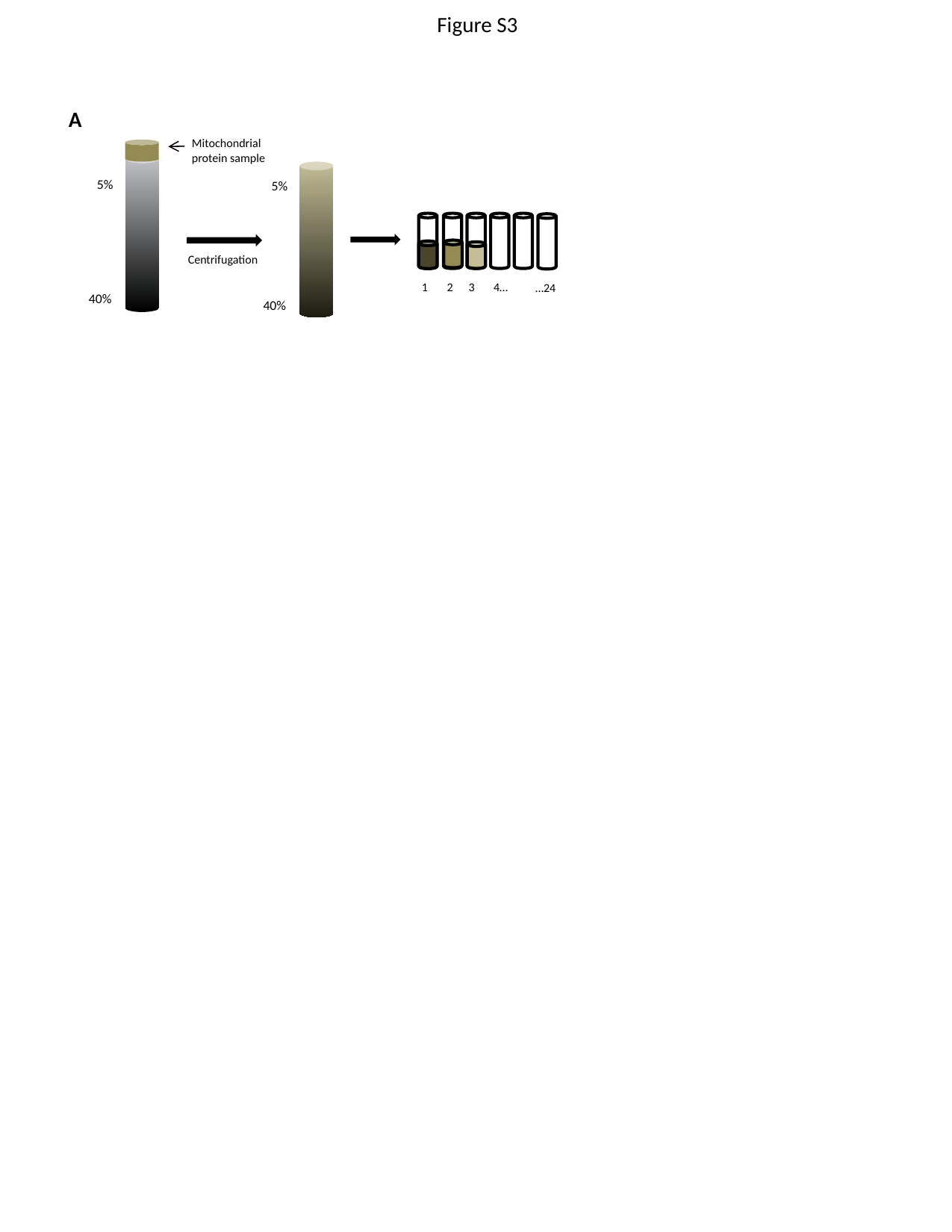

Figure S3
A
Mitochondrial protein sample
5%
40%
5%
40%
1
2
3
4…
…24
Centrifugation

## Slide 4
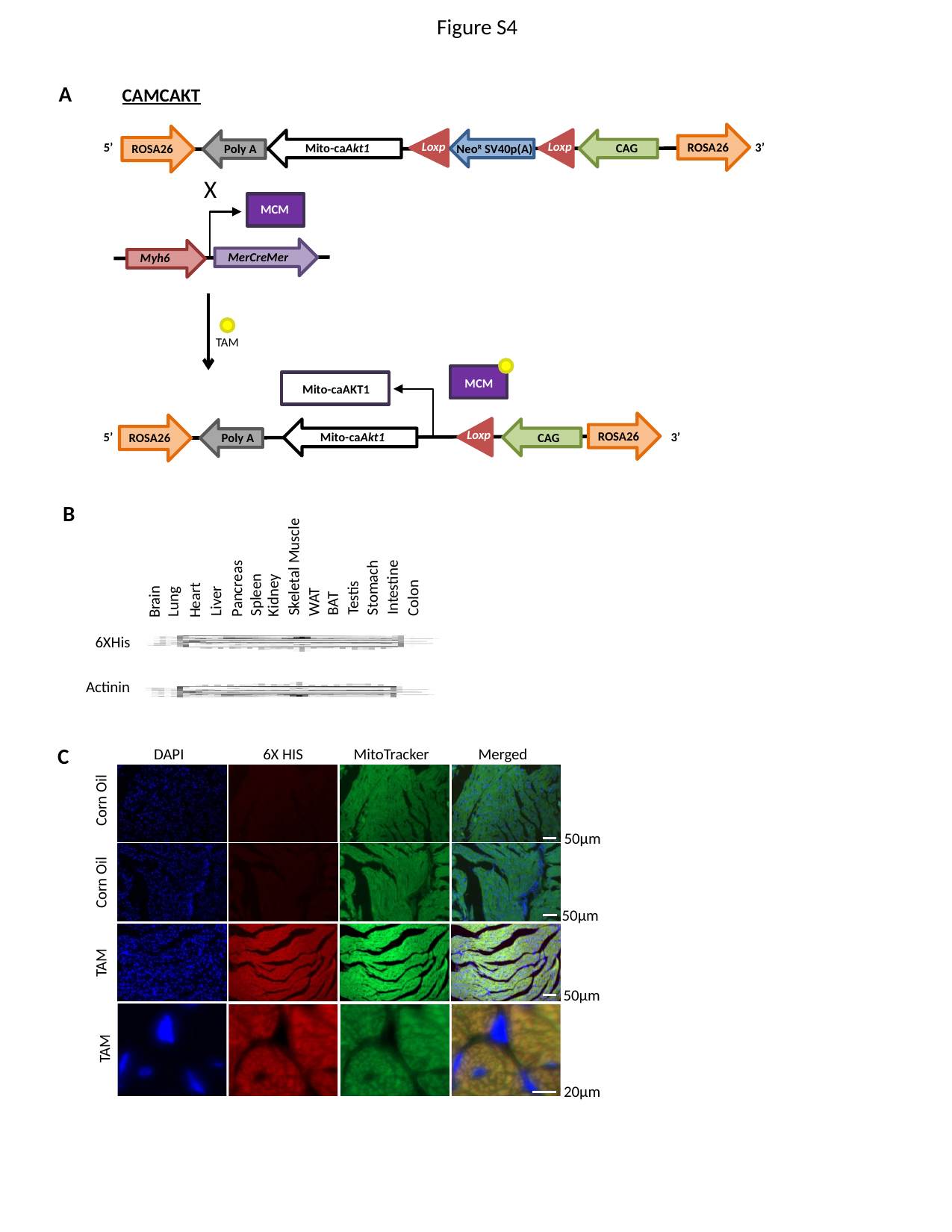

Figure S4
A
CAMCAKT
Loxp
5’
3’
Poly A
ROSA26
ROSA26
Loxp
CAG
Mito-caAkt1
NeoR SV40p(A)
X
MCM
MerCreMer
Myh6
TAM
MCM
Mito-caAKT1
Loxp
3’
5’
Poly A
ROSA26
ROSA26
Mito-caAkt1
CAG
B
Skeletal Muscle
Intestine
Stomach
Pancreas
Spleen
Kidney
Colon
Testis
Heart
Liver
WAT
Brain
Lung
BAT
6XHis
Actinin
C
DAPI
6X HIS
MitoTracker
Merged
20μm
Corn Oil
Corn Oil
TAM
TAM
50μm
50μm
50μm

## Slide 5
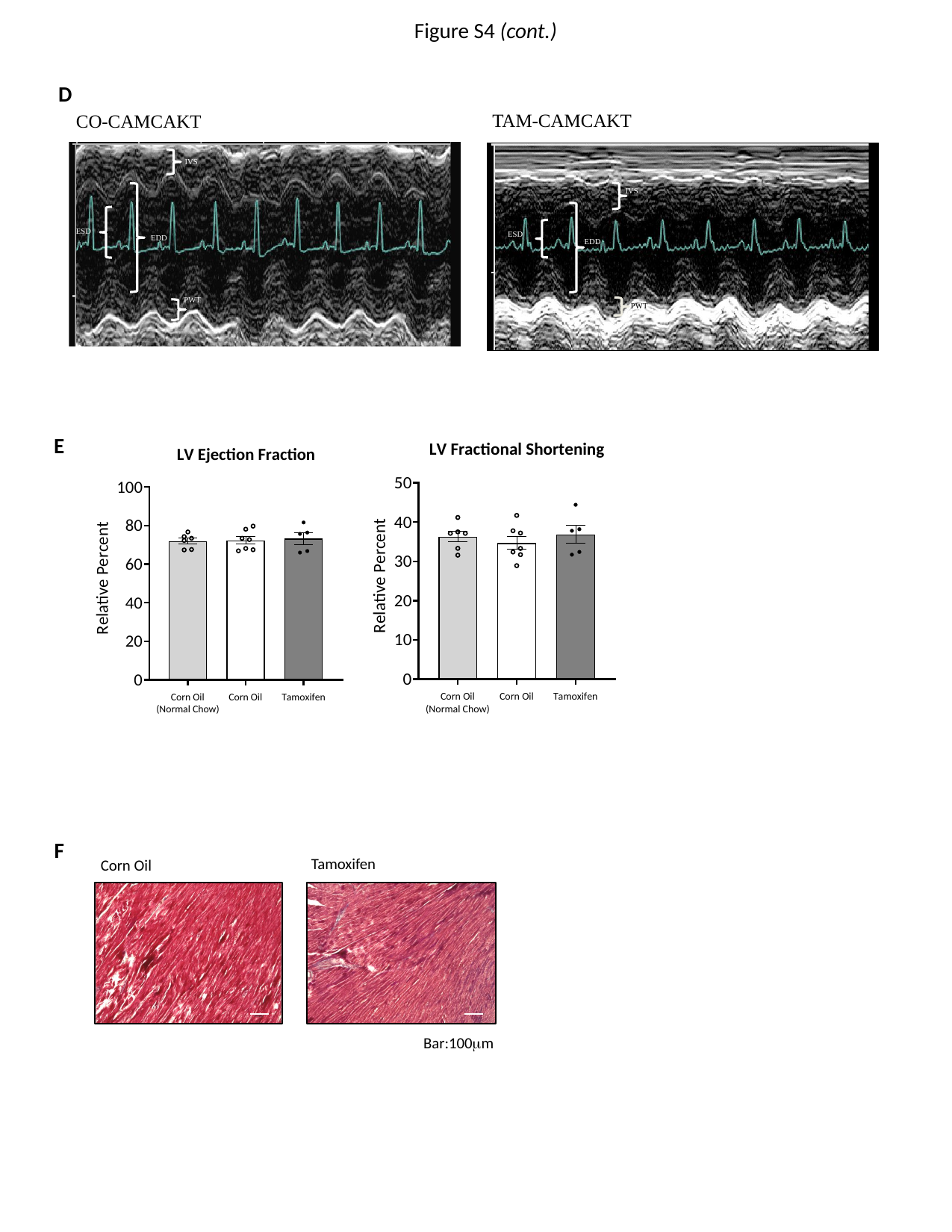

Figure S4 (cont.)
D
TAM-CAMCAKT
IVS
ESD
EDD
PWT
CO-CAMCAKT
IVS
ESD
EDD
PWT
E
F
Tamoxifen
Corn Oil
Bar:100μm

## Slide 6
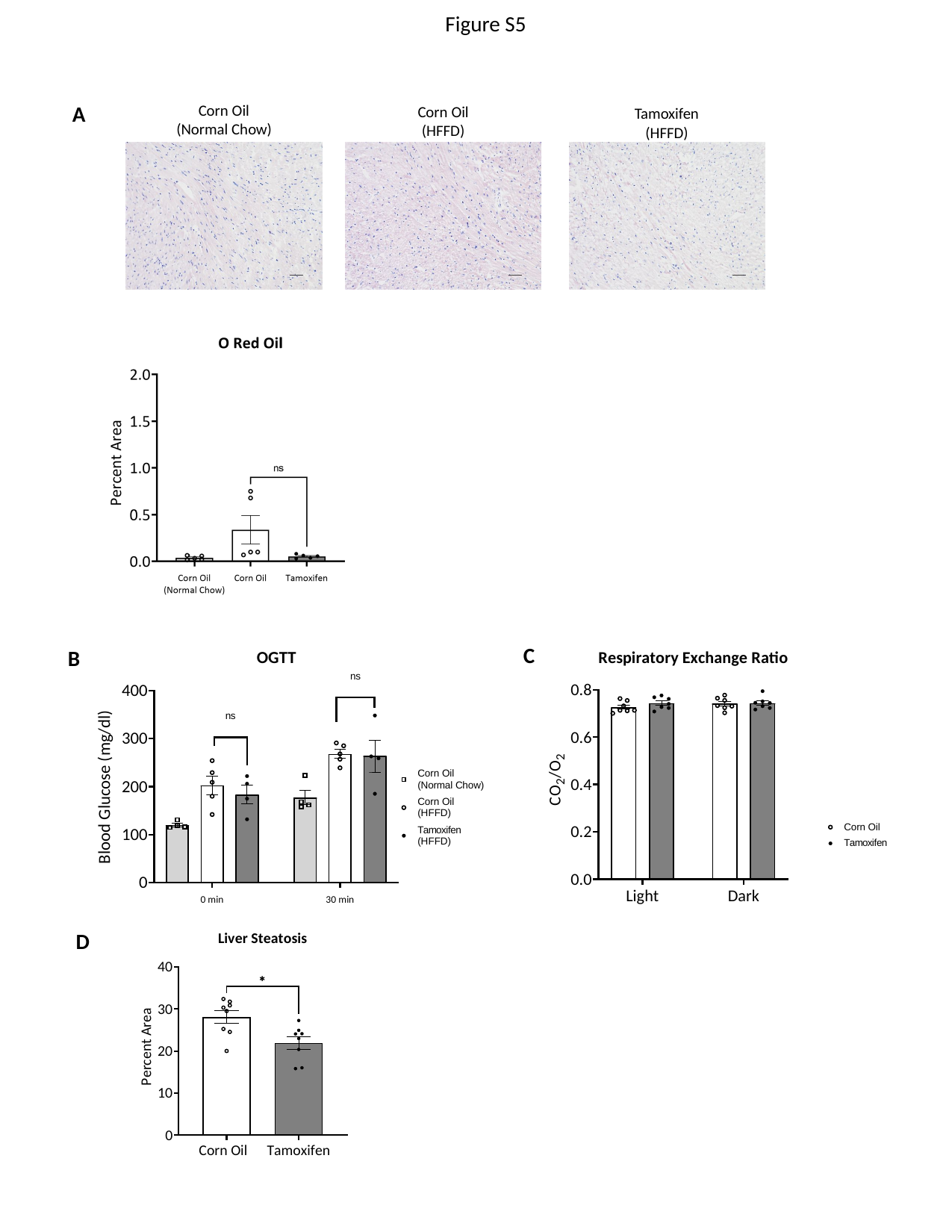

Figure S5
A
Corn Oil
(Normal Chow)
Corn Oil
(HFFD)
Tamoxifen
(HFFD)
C
B
D
