# Supplementary material for: Dual roles of myocardial mitochondrial AKT on diabetic cardiomyopathy and whole body metabolism
Source: Cardiovasc Diabetol. 2023 Oct 27;22:294. doi: 10.1186/s12933-023-02020-1 (PMC10612246; doi:10.1186/s12933-023-02020-1)
Supplement: Supplementary file 2 — Additional file 2. Further information of the experimental methods. [file 12933_2023_2020_MOESM2_ESM.docx]

**Supplemental Methods**

Study Approval

The experimental protocols were approved by the Institutional Animal Care and Use Committee at the City of Hope National Medical Center (20048) and the University of California at Irvine (AUP-18-113). All experiments were performed in accordance with federal and local guidelines.

Immunofluorescence Microscopy

Paraffin-embedded heart sections were deparaffinized with xylene and rehydrated with graded ethanol. For antigen retrieval, the slides were immersed in 0.1M Tris (pH10) buffer and heated with an 1100W GE microwave oven for three 3-min cycles at power levels of 5, 4 and 3. After cooling, the slides were rinsed with PBS. The tissue sections were circled with a liquid Blocker Super Pap Pen before incubating in anti-6x-His-Tag antibody (Cell Signaling Technologies, 12698S) (diluted in 1x PBS, 4% BSA, 0.1% Triton X-100) and incubated overnight in a humified chamber at 4°C. After extended washes with PBS, fluorescence-conjugated secondary antibodies were applied and placed in a humidified chamber, incubated in a GE microwave oven at power level 4 for 3 minutes, rinsed with PBS and stained with Mitotracker Green (10nM) and DAPI (1μg/ml) for 20 minutes at room temperature. After washing with PBS, the slides were mounted for imaging with a Keyence BZ-X810 Inverted Microscope.

Mitochondria Isolation from Cardiac Tissue

Freshly collected heart tissue was minced and rinsed in PBS. Tissue was resuspended in RBS buffer (1X SIGMAFAST^TM^ protease inhibitor (Sigma, S8830), 5mM KCl, 1mM MgCl­_2_, 20mM HEPES pH 7.0) and incubated on ice for 10 minutes before being ground using a Dounce homogenizer – 25 passes with a loose pestle and 25 passes with a tight pestle. The solution was then centrifuged at 500g for 5 minutes. The mitochondria containing supernatant was then centrifuged for 30 mins at 14,000g. The resultant mitochondrial pellet was rinsed and resuspended in RBS buffer to approximately 1 mg/mL. Mitochondrial protein concentration was determined by Bradford Assay (Bio-Rad, 5000006) according to manufacturer’s standard protocol.

Western Blotting

Mitochondrial fractions were dissolved in 2% lauryl maltoside solution supplemented with 10% SigmaFAST^TM^ protease inhibitor (Sigma-Aldrich, S8820). Protein concentrations were determined with an Eppendorf BioPhotometer by Bradford assay. Equal amounts of proteins from each sample were resolved with 10% SDS-polyacrylamide gel and then transferred onto polyvinylidene difluoride membranes. The membranes were blocked with 5% fat-free milk or 5% BSA for one hour before incubation with primary antibodies overnight at 4°C, washed three times with TBS-T (20 mM Tris–HCl, pH 7.5, 0.5mM NaCl, and 0.1% Tween 20), incubated with anti-rabbit IgG, horseradish peroxidase-linked antibody (Cell Signaling, 7074) (1:2000 dilution in 5% fat-free milk or 5% BSA), washed three times with TBS-Tween, and then incubated with West Pico Chemiluminescent Substrate to visualize the proteins (ThermoFisher Scientific, 34580). The images were acquired with a Syngene G:BOX and analyzed with ImageJ.

ATP Kinase Assay

Mitochondria preparations were isolated as described above and AKT enzymatic activity was analyzed with an AKT Activity Assay Kit (Abcam, ab65786) according to the manufacturer’s instructions. AKT protein was immunoprecipitated from the solubilized mitochondria preparations and the enzymatic activities were quantitated with recombinant GSK-3α protein. Phosphorylation of GSK-3α was visualized by western blot using anti-phospho-GSK-3α antibodies.

RNA Isolation

30-50μg of tissue was used per sample for RNA isolation. Tissue samples were rinsed in PBS and homogenized in 700μL TRIzol™ (Invitrogen, 15596026) using a plastic microfuge tube pestle. Samples were incubated at room temperature for 5 minutes before adding 200μL chloroform and vortexed. After, samples were incubated for 3 minutes at room temperature before centrifugation at 12000g for 15 minutes at 4°C. The resultant top aqueous layer was transferred to a new tube and 500μL isopropanol added, mixed and incubated at room temperature for 10 minutes. Samples were then centrifuged at 12000g for 15 minutes at 4°C. The resulting pellet was washed then with 500μL ice cold 80% ethanol and then centrifuged again at 10,000g for 10 minutes at 4°C. The supernatant was discarded, and the pellet was dried for 5 minutes and then resuspended in 20-50μL DEPC water.

Masson’s Trichrome Staining

Tissue samples were fixed overnight in 10% buffered formalin at 4°C, washed twice with phosphate-buffered saline (PBS), incubated in 65% and 70% ethanol, progressively dehydrated with graded ethanol and Histoclear with a Leica TP1020 tissue processor, and embedded in paraffin. 4μm paraffin sections were deparaffinized in xylene and rehydrated in graded ethanol. The sections were mordanted in Bouin’s solution (picric acid, formaldehyde, and acetic acid) overnight. After washing, they were stained with Weigert’s Iron Hematoxyline Solution for 30 min, followed by Biebrich Scarlet-Acid Fucshin for 15 min, phosphomolybdic-phosphotungstic acid solution for 10 min, and then aniline blue for 20 min. After brief submersion in 0.5% acetic acid, the slides were dehydrated with 95% alcohol, 100% alcohol, and xylene. Images were captured with a Zeiss AxioPlan2 microscope and analyzed with AxioVision Rel 4.6 software or with a Keyence BZ-X810 Inverted Microscope and analyzed with Keyence BZ-X800 Analyzer software. Blue coloration of collagen in the extracellular matrix (ECM), was quantified with ImageJ.

Echocardiogram

Mice were screened by echocardiography at the Mouse Physiology Core Laboratory at the UCLA Department of Physiology. Cardiac function was evaluated by noninvasive ultrasound echocardiography under light isoflurane sedation (0.5–1.0%) to prevent movement and cardiodepression. Data were acquired using a two-dimensional-guided M-Mode and spectral Doppler imaging with a Siemens Acuson Sequoia Model C256 equipped with a 15 MHz probe (Siemens Medical Solutions, 15L8). Mice were evaluated in diastole to obtain heart dimension and function measurements.

M-mode was used to measure EDD, ESD, IVST, PWT. From these values, we obtained the LVFS,  LVEF ( Ejection fraction is measured directedly by the echo machine following Simpson's rule from the short axis). The VCF is obtained from the M-mode in conjunction with pulse wave doppler and calculated as LVFS/Aortic ejection time. LVFS and VCF are indices of contractility and LVEF is the volumetric fluid ejected from the LV chamber during contraction. The mass of the left ventricle as a relative percent to total heart mass was calculated as: LV_mass_ = ((VST+EDD+PWT)^3^ – EDD^3^)*1.055 (VST – Ventricle Septum Thickness, EDD – End Diastolic Diameter, PWT – Posterior Wall Thickness, Mass Correction Value = 1.055). M mode is a time-tested method of measuring LV volume but may have limited precision for very small animals.

qPCR

RNA was reverse transcribed into cDNA using an iScript gDNA Clear cDNA Synthesis Kit (Bio-Rad, 1708891) according to the manufacturer’s protocol. Quantitative RT-PCR was performed using Apex qPCR GREEN Master Mix (Genesee Scientific, 42-119PG). 300ng of cDNA was used as input in a 10μL total reaction volume on a LifeTechnologies QuantStudio 6 RT-PCR instrument. The qPCR program consisted of an initial activation at 95°C for 10 min, followed by 40 cycles of 95°C for 15s and 60°C for 1 min. 36B4 was used as an endogenous control for normalization. 36B4: (forward: 5′- ACTGGTCTAGGACCCGAGAAG -3′; reverse: 5′- TCAATGGTGCCTCTGGAGATT -3′), PGC1α: (forward: 5'-CTCTCAGTAAGGGGCTGGTT-3'; reverse: 5'-AGCAGCACACTCTATGTCACTC-3') PPARα: (forward: 5'-GCAGCTGTTTTGGGGGCT-3'; reverse: 5'-TCAACTTGGCTCTCCTCTAAGT-3').

Mitochondrial DNA Content

Quantitative PCR was performed using Apex qPCR GREEN Master Mix (Genesee Scientific, 42-119PG). 5ng of DNA was used as input in a 20μL total reaction volume on a LifeTechnologies QuantStudio 6 RT-PCR instrument. The qPCR program consisted of an initial activation at 95°C for 10 min, followed by 40 cycles of 95°C for 15s and 60°C for 1 min. β-globin was used as a nuclear DNA control for normalization. COX-II:(forward: 5′-GCCGACTAAATCAAGCAACA-3′; reverse: 5′-CAATGGGCATAAAGCTATGG-3′), β-globin: (forward: 5′-GAAGCGATTCTAGGGAGCAG-3′; reverse: 5′-GGAGCAGCGATTCTGAGTAGA-3′).

Analysis of Mitochondrial O_2_ Respiration by Metabolic Flux Measurement

To measure mitochondrial function in cells, a Seahorse Bioscience XFe24 Extracellular Flux Analyzer was utilized according to the manufacturer’s protocol. Mitochondria were isolated from heart tissue as described previously. 10μg of mitochondrial protein was plated onto each experimental well of a Seahorse XFe24 assay plate by centrifugation at 3000rpm for 20 minutes. The wells were then filled to 500μL with 1X Mitochondrial Assay Solution (MAS) (The following solutions were used: 70mM sucrose, 220mM mannitol, 10mM KH_2_PO_4_, 5mM MgCl_2_, 2mM HEPES, 1.0mM EGTA and 0.2% (w/v) fatty acid-free BSA, pH7.2). Coupling assay metabolic substrate conditions were 10mM Pyruvate/5mM Malate. After incubation at 37°C for 8 minutes, the assay plate was loaded into the XFe24. Two baseline measurements of oxygen consumption rate (OCR) were taken before sequential injection of ADP and mitochondrial inhibitors of final concentrations: ADP (40mM), oligomycin (2.5µg/ml), carbonilcyanide p-triflouromethoxyphenylhydrazone (FCCP) (4µM) and rotenone (0.1µM). Two measurements were taken after the addition of each inhibitor. OCR values were automatically calculated and recorded by the Seahorse Wave Controller software.

ATP Quantification

Mouse hearts were collected after euthanization and snap-frozen in liquid nitrogen. Metabolites were extracted with 80% methanol and quantified with mass spectrometry. Extracted metabolites in 80% methanol were dried to a pellet in a vacuum centrifuge at 30℃. ATP identification was conducted using MALDI mass spectrometry. The dried metabolite pellet was reconstituted in 50% acetonitrile (0.1% TFA) and mixed with α-Cyano-4-hydroxycinnamic acid (CHCA) matrix at a 1:1 ratio. The mixture was then spotted on the plate. The mass spectrum was generated using a SCIEX TOF/TOF 5800 System and TOF/TOF Series Explorer software. Operating mode was set as MS Reflector Negative Default and under automatic acquisition control. Mass detecting range was 50 to 1000Da with a focus mass at 500Da. Total shot per spectrum was 3000. Continuous stage motion mode was set at 600μm/second. Search pattern parameters were set as random uniform. Laser intensity was fixed at 3350. Digitizer bin size was 0.5ns with 1000MHz input bandwidth. Detector voltage multiplier was 0.59 and the final detector voltage was 1.770. Laser pulse rate was set at 400Hz. Metabolite peaks were identified based on their mass-to-charge (m/z) ratio referenced from multiple Massbank databases (MoNA <http://mona.fiehnlab.ucdavis.edu/>, MassBank <http://www.massbank.jp/?lang=en>, and NORMAN MassBank <http://massbank.eu/MassBank/> ) and quantified by their peak intensities. The average peak intensities for each metabolite were calculated from 7 mass spectra generated from the same sample and presented as relative fold change to the control group. The peak intensity of CHCA (m/z=188) matrix was used as signal normalization control for sample loading and excitation efficiency.

TEM

After euthanization and perfusion, mouse hearts were harvested and immersed in 4% glutaraldehyde in PBS overnight at 4°C. The ventricles were further excised and trimmed into cubes ~1 mm^3^. After primary fixation with 2% PFA/2.5% Glutaraldehyde/0.1M cacodylate for 2 hours at 4°C and post fixed with 1% osmium tetraoxide/0.1M Cacodylate for 2 hours at 4°C, the ventricular cube were incubated in 0.25% uranyl acetate at 4°C overnight. The tissue was then dehydrated with gradient ethanol and embedded in Epon resin. Ultrathin sections were cut and placed on copper grids, followed by staining with uranyl acetate and lead citrate. Samples were then imaged at UC Irvine CTEM using a Philips CM10 transmission electron microscope. The magnification in images of tissue sections and single mitochondrion are X1100 and X11000 respectively.

MicroPET-CT Imaging and Biodistribution

The microPET-CT studies were carried out on a SOFIE GNEXT PET/CT which is a high performance and high flexibility preclinical *in vivo* imaging system with anesthesia systems. Briefly, a needle catheter was inserted into a lateral male mouse tail vein after (fasted for 6 hours) anesthetization with 2 - 4% isoflurane in oxygen. Mice were transferred into the PET-CT scanner before injection of 7.4 MBq [^18^F] Fluoro-4-Thia-Oleate ( [^18^F]FTO) in 1% serum albumin with saline. 35 min dynamic microPET scans were conducted, followed by a 1 min CT scan. The images were reconstructed by three-dimensional ordered subsets expectation-maximization (3DOSEM) using the integrated GNEXT Acquisition Engine software. Co-registered PET/CT images were post-processed with Amide’s a Medical Imaging Data Examiner (AMIDE) software.

The biodistribution of [^18^F]FTO was quantified at the end of PET-CT scans for all mice. Mice were euthanized and organs/tissues of interest (blood, heart, lung, liver, gallbladder, spleen, stomach, kidneys, pancreas, duodenum, small intestine, cecum, large intestine, brown fat, muscle, bone, and brain) were harvested. Organs/tissues were weighted and counted in a Wizard2 gamma counter (PerkinElmer Health Sciences Inc., Shelton, CT, USA) with three tubes containing 1% of the injected dose (standards) and three empty tubes (background). Radioactive uptake were calculated and reported as percentage injected dose per gram of sample mass (% ID/g) or per of whole organ weight (%ID/organ)[1].

Oil Red O Staining

Frozen heart sections were stained using an Oil Red O Stain kit according to the manufacturer’s instructions (Statlab, ktoro).

Serum Fatty Acid Quantification

Serum was collected from mice after five hours of fasting. Serum levels of free fatty acid were measured using a Free Fatty Acid Assay kit according to the manufacture’s instructions (Abcam, ab65341).

Metabolic Cage

Energy expenditure was evaluated using a TSE PhenoMaster System. Mice were singly housed in the metabolic cages and provided HFFD. Movement, CO2 production and O2 consumption were collected at 30 min intervals. The light cycle ran from 6 AM to 6 PM followed by a dark cycle. Measurements are normalized to mice weights at the start of the experiment. Mice were allowed to acclimate to the metabolic cage for 24 hours before calculated measurements.

Body Composition

Body composition was determined using an Echo Medical Systems EchoMRITM 3-in-1 body composition analyzer. Mice were individually placed in acrylic specimen holders based on body size and compressed in place and placed into the EchoMRI^TM^ analyzer for a live reading. The analyzer was calibrated using a corn oil cartridge. Following the manufacturer’s standard protocol, measurements of whole-body fat, lean mass, free water and total water, and total weight were taken. Calculation of body fat percentage is the reported as whole body fat mass divided by the total weight. Lean mass percent is reported as the lean mass divided by the total weight.

Liver Steatosis Quantification

Liver tissue sections were prepared and H&E stained. 10 images at 20x were captured by random sampling with a Zeiss AxioPlan2 microscope. Each image was then processed by ImageJ to determine the degree of steatosis. White areas were quantified using the color threshold function in ImageJ. White pixels were counted by setting the following threshold settings: Hue (0, 255), Saturation (0, 30) and Brightness (0, 255). Pixels under threshold were measured and considered as an area of lipid droplet. The area of lipid droplets is then divided by total captured area giving a percentage of steatosis coverage.

Co-Immunoprecipitation

Co-IPs were performed with cardiac mitochondrial lysate from mice using AKT1 antibody (Abcam, Ab32038). Soluble mitochondrial lysate were obtained using 1x PBS with 0.1% Lauryl Maltoside. 10μg of AKT1 antibodies were crosslinked to magnetic beads using Pierce Crosslink Magnetic IP/CO-IP kit (ThermoFisher, 88805) and used in the immunoprecipitation assay. (ATP5A1, Abcam ab14748; ATPB, Abcam ab14730; ATP Synthase Gamma, Genetex GTX114275; ATP Synthase B1, Genetex GTX102980; ATP Synthase C, Abcam ab181243)

Sucrose Gradient Analysis

10μg of isolated mitochondria were lysed with 100μl of 1x PBS with 0.1% Lauryl Maltoside. After centrifugation, the soluble fractions were loaded onto a linear 5-40% sucrose gradient in a buffer of 10mM Tris (pH7.5), 100mM NaCl, 0.1% NP40 with protease inhibitors. After ultracentrifugation at 100,000g for 16.5 hours at 4^o^C, the fractions were collected using a 27-Guage needle from the bottom of the tube. A total of 24 0.5ml fractions were collected from each sample and the even-numbered fractions were used in the western blot analysis for complex V components. (ATP5A1, Abcam ab14748; ATPB, Abcam ab14730; ATP Synthase Gamma, Genetex GTX114275; ATP Synthase Delta, Genetex GTX101503; ATP Synthase B1, Genetex GTX102980; ATP Synthase C, Abcam ab181243)

**Supplemental Methods References**

[1] Li J, Hu W, Peng J, Wong P, Kandeel F, Olafsen T, et al. Heart Uptake of [(18)F]Fluoro-4-Thia-Oleate in a Non-Alcoholic Fatty Liver Disease Mouse Model. Pharmaceuticals (Basel). 2022;15.

[2] Chen Y, Craigen WJ, Riley DJ. Nek1 regulates cell death and mitochondrial membrane permeability through phosphorylation of VDAC1. Cell Cycle. 2009;8:257-67.
